# Storage Buffer Composition Impacts Internal Structure, Freeze–Thaw Stability, and Transfection Efficiency of mRNA-Lipid Nanoparticles

**DOI:** 10.1021/acsnano.5c22170

**Published:** 2026-06-12

**Authors:** Meysam Mohammadi-Zerankeshi, Dylan J. Charland, Keira A. Donnelly, Geoffrey T. Nash, Jiale Shi, Dipak N. Patil, Julia Ennis, Kenneth G. Rodriguez, Sonia Corba, Noah A. Wambolt, Haocheng Chueh, Khaled AboulFotouh, Mohammed R. Kawelah, Younghoon Oh, Dennis Yang, Ken K. Qian, Qiang Cui, Keith P. Johnston, Daniel A. Estabrook, Alexander E. Marras

**Affiliations:** 1 Walker Department of Mechanical Engineering, 12330 University of Texas at Austin, Austin, Texas 78712, United States; 2 1539 Eli Lilly and Company, Indianapolis, Indiana 46225, United States; 3 Department of Biomedical Engineering, 12330 University of Texas at Austin, Austin, Texas 78712, United States; 4 Department of Chemistry, 1846 Boston University, Boston, Massachusetts 02215, United States; 5 Eurofins PSS Insourcing Solutions, LLC, Lancaster, Pennsylvania 17601, United States; 6 McKetta Department of Chemical Engineering, 12330 University of Texas at Austin, Austin, Texas 78712, United States; 7 Lilly Seaport Innovation Center, Boston, Massachusetts 02210, United States

**Keywords:** RNA delivery, mRNA lipid nanoparticles, SAXS, molecular dynamics, ionizable lipids, buffer effects, formulation development

## Abstract

Messenger RNA (mRNA) lipid nanoparticles (mRNA-LNPs) are central to emerging vaccines and therapeutics, but their wide implementation is constrained by limited endosomal escape and instability during long-term storage and freezing. While buffers are routinely optimized to prevent instability, the impact of buffer on the internal structural organization of LNPs and, consequently, their delivery efficiency remain unresolved. Here, we study the impact of storage in Tris, histidine, and citrate buffers for mRNA-LNPs formulated with LP-01, MC3, and SM-102 ionizable lipids. We demonstrate that storage buffer identity and concentration govern mRNA-LNP internal ordering before and after freeze–thaw and are thus critical parameters for engineering high-performance formulations. Deconvoluting ordered phases into an mRNA-lipid region and excess lipid region reveals the importance of excess ionizable lipid behavior in enhancing endosomal escape. Prior to freezing, citrate buffer enhances transfection efficiency by promoting a transition to the fusogenic inverse hexagonal (H_II_) phase earlier during acidification, facilitated by a greater amount of ordered excess ionizable lipid. In contrast, Tris buffer provides the highest transfection efficiency after freeze–thaw by preventing aggregation and cargo loss while promoting favorable internal structure. Increasing Tris concentration from 10 to 50–150 mM leads to mRNA-rich bleb formation in freeze–thawed mRNA-LNPs, which improves freeze–thaw stability and thus transfection efficiency by mitigating mRNA-lipid adduct formation and accommodating a larger excess ionizable lipid region to facilitate H_II_ phase formation. These findings establish a direct structural link between buffer conditions, particle size, internal morphology, and transfection efficiency, highlighting the importance of buffer composition in modulating mRNA-LNP performance.

## Graphical abstract

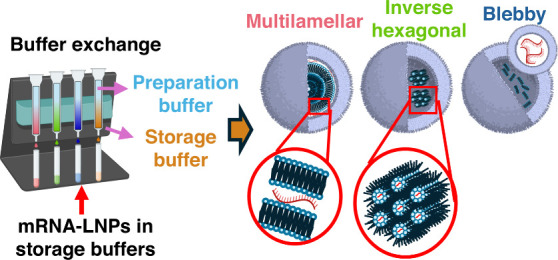

Messenger RNA (mRNA) has broad potential for treating refractory diseases requiring protein expression, including potential clinical applications in protein replacement therapy, cancer immunotherapy, gene editing, tissue engineering, and vaccination.^1–3^ mRNA therapies may achieve higher therapeutic efficacy by enabling *in vivo* expression of proteins for an extended duration compared to direct administration of traditional proteins, which are rapidly cleared. Additionally, mRNA transcripts exhibit relatively high transfection efficiency, pose no risk for mutagenesis, and have high modularity along with scalable manufacturing capabilities. However, the broad clinical application of mRNA is limited by its poor stability in systemic circulation and reduced cellular internalization. As such, there is a need for an efficient drug delivery system that can protect mRNA from RNase-induced degradation and enhance its cellular uptake and endosomal release to the cytosol, where translation into the target protein is initiated. Lipid nanoparticles (LNPs) are the most clinically advanced platform for mRNA delivery, validated by the U.S. Food and Drug Administration (FDA)-approved COVID-19 vaccines^4–6^ and, more recently, for respiratory syncytial virus (RSV) prevention.^7^

LNPs are composed of an ionizable lipid, polyethylene glycol (PEG)-anchored lipid (PEG-lipid), cholesterol, and an amphiphilic phospholipid (Figure 1a).^8^ The ionizable lipid undergoes protonation at low pH (below lipid p*K*_a_), enabling complexation with anionic RNA during assembly and driving pH-dependent structural transitions critical for fusogenesis to promote endosomal escape.^9^ Cholesterol, phospholipids, and PEG-lipids enhance the membrane integrity, cellular uptake and particle stability, respectively.^10,11^ Among all components, the ionizable lipid has received the greatest attention with regard to potency, mRNA distribution, and degradability of mRNA-LNPs.^12^ Rapid LNP degradation after mRNA delivery, facilitated by hydrolyzable ester linkages, helps minimize excessive inflammation linked to vaccine reactogenicity.^13^

**Figure 1:**
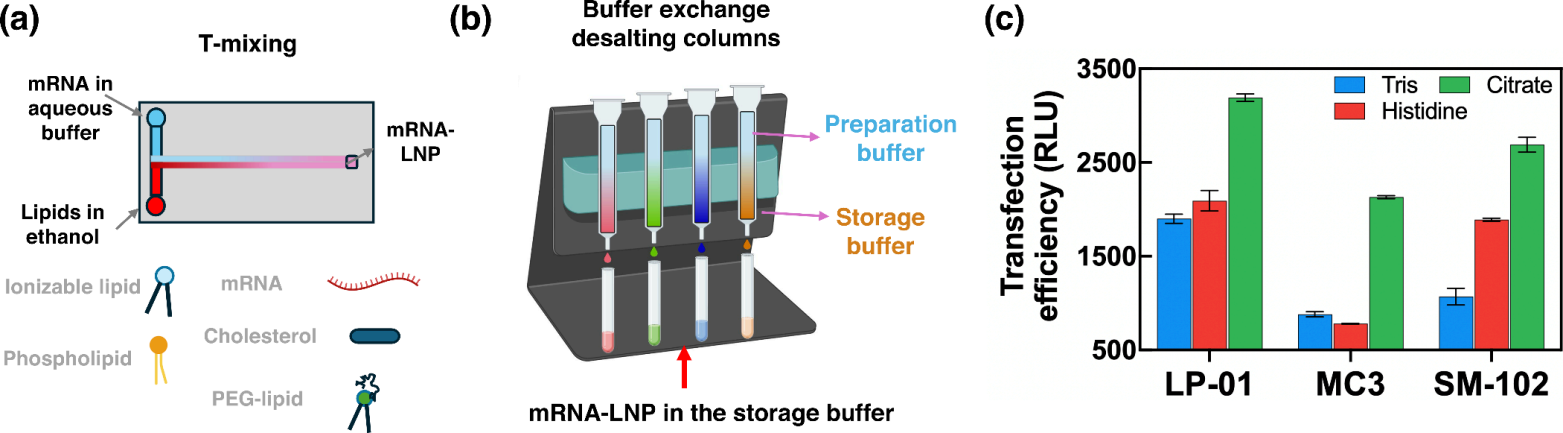
mRNA-LNP synthesis and the influence of storage buffer. (a) T-mixing technique is used to fabricate FLuc mRNA-LNPs. (b) PD-10 columns are used to exchange into different storage buffers of interest. (c) FLuc mRNA-LNP transfection efficiency results for different ionizable lipids in Tris, histidine and citrate storage buffers in HepG2 cells.

Despite the successful development of mRNA-LNPs, a major limitation remains that over 90% of the RNA cargo becomes trapped in endosomes and lysosomes, posing a significant barrier to effective delivery.^14–16^ To enhance endosomal escape, many studies have examined the relationship between LNP internal structure and performance.^9,12^ Notably, lipid phase transitions to inverse hexagonal (H_II_) or cubic phases during endosomal acidification have been shown to increase fusogenicity with endosomal membranes and promote RNA release.^17–20^ While lipid composition and chemistry are well-studied determinants of these transitions,^21^ less attention has been given to the role of formulation and storage buffers. We have recently shown that employing a mildly acidic, histidine-containing buffer can enhance siRNA-LNP stability and bioactivity at room temperature by mitigating RNA-lipid adduct formation.^22^ Tang et al.^23^ demonstrated that the pH of the RNA acidifying buffer, rather than the salt type or concentration, significantly affects both the encapsulation efficiency and protein expression of SM-102 mRNA-LNPs, with pH 4 achieving optimal results. By contrast, different storage buffers did not significantly influence structure and performance. Similarly, Cheng et al.^24^ reported that use of highly concentrated, acidic citrate buffers during KC2 mRNA-LNP formation improved transfection efficiency and was correlated with an increased number of empty blebs within the particle, although the authors acknowledged that the exact mechanism for this improvement was unclear. By contrast, Binici et al.^25^ found that increasing citrate buffer molarity to 300 mM did not alter the structure or potency of SM-102 mRNA-LNPs. A recent study showed that the RNA acidifying buffer consistently influenced the bulk phase transitions and performance of mRNA-LNPs formulated with DLin-MC3-DMA (MC3), SM-102 or ALC-0315.^26^ In this study, citrate buffer facilitated an earlier and more efficient transition to a stabilized inverse hexagonal phase compared to phosphate or acetate buffers, which in turn enhanced endosomal escape efficiency. Even more recently, a control strategy with two impingement jets in series was presented to independently control LNP size, morphology, and bleb content.^27^ Blebs are protruding structures containing an aqueous core, with or without mRNA, enclosed by a lipid bilayer and phase-separated from a denser lipid-rich core, which likely contains ionizable lipids. The bleb bilayer is thought to be enriched in bilayer-forming components such as DSPC, cholesterol, and PEG-lipids. Formation of such blebbed-like mRNA-LNPs positively correlated with transfection efficiency, consistent with the findings of Cheng et al.^24^

Beyond the influence on pH-dependent phase transitions, storage buffers also play a key role in the freeze–thaw stability of mRNA-LNPs.^28,29^ Freezing is often required in long-term storage, as subzero temperatures protect mRNA from degradation and leakage.^30^ However, freeze–thaw cycles can induce aggregation, particle rupture, and mRNA release due to ice crystallization and osmotic stress.^31,32^ As such, cryoprotectants like sucrose are commonly added to the drug product matrix to minimize these instabilities.^33,34^ Henderson et al.^28^ found greater stability of mRNA-LNPs in Tris and HEPES buffers relative to phosphate-buffered saline (PBS), suggesting that these buffers themselves can function as cryoprotectants. Moderna’s SpikeVax uses Tris as the storage buffer, while Pfizer/BioNTech’s COMIRNATY formulation was switched from PBS to Tris storage buffer to enhance the stability of mRNA-LNPs in frozen storage. Such decisions demonstrate the significance of storage buffer selection in the long term stability of mRNA-LNPs.^35,36^

Despite the critical function of the storage buffer, the mechanistic role of buffer on the internal structure of mRNA-LNPs both before and after freeze–thaw storage remains elusive. Herein, we systematically investigate three common buffers (Tris, histidine, and citrate) along with three widely studied ionizable lipids (LP-01, MC3, SM-102) to develop rational guidelines for engineering internal LNP structures that maximize transfection potency. All mRNA-LNPs were formulated using a consistent RNA acidifying buffer, then buffer exchanged into the storage buffer of interest (Figure 1b). Transfection efficiency was studied in four human cell lines: HepG2 (hepatocellular carcinoma), HEK293T (embryonic kidney), Jurkat (immune T-cell lymphoma) and HeLa (cervical carcinoma). Structural characterization was performed by small-angle X-ray scattering (SAXS), cryogenic transmission electron microscopy (cryo-TEM) as well as molecular dynamic (MD) simulations. To aid elucidation of these structurally complex mRNA-containing LNPs, control experiments were also performed with empty-LNPs, which exhibit well-defined, pH-dependent phase transitions readily identified with SAXS.^12,37^

We demonstrate that buffer identity plays a critical role in mRNA-LNP internal structure and, subsequently, transfection efficiency. For refrigerated mRNA-LNPs, citrate buffers yielded the highest transfection efficiency by promoting earlier pH-dependent transitions to the inverse hexagonal phase within excess ionizable lipid domains. However, Tris emerged as a superior buffer for frozen mRNA-LNPs, consistent with its favored use for COVID-19 vaccines.^35^ We provide mechanistic descriptions of why Tris was the only buffer to maintain particle morphology and internal structure for high transfection efficiency after freeze–thaw. Cellular trafficking assays confirm that buffer composition influences uptake and cargo delivery, underscoring the impact of buffer on drug product potency. Notably, increasing Tris concentration of freeze–thawed mRNA-LNPs from 10 to 50 mM and higher enhances drug product potency by promoting mRNA-rich bleb formation through weakening mRNA-ionizable lipid interactions, as hinted by MD simulations. Collectively, these mechanistic insights highlight the importance of buffer design in optimizing critical quality attributes, particularly internal structure, throughout mRNA-LNP manufacturing and frozen storage to support therapeutic performance.

## Results and Discussion

### Effect of Buffer Species on FLuc mRNA-LNP Critical Quality Attributes (CQAs)

To study the effect of storage buffer species on mRNA-LNP CQAs (*i.e.*, hydrodynamic diameter, encapsulation efficiency, and transfection efficiency), a consistent LNP formulation was exchanged into three storage buffers: Tris (p*K*_a_ ≈ 8.1), histidine (p*K*_a_ ≈ 6.0) and citrate (p*K*_a_ ≈ 4.8) (Figure 1b).^38^ Formulations using LP-01, MC3 and SM-102 ionizable lipids were compared for all three buffers at 50 mM buffer, 45 mM NaCl and 5 wt % sucrose. The mRNA-LNPs formulated in Tris and histidine buffers achieved the target pH of 7.4, while those in citrate buffer displayed a modestly reduced pH of 6.9. At these measured pH values, all formulations exhibited high encapsulation efficiency (89–94%), as measured by the RiboGreen assay. Dynamic light scattering (DLS) and cryo-TEM revealed particle sizes in the range of 60–120 nm (Figure S1). Cryo-TEM also showed that particles were generally spherical and monodisperse, although histidine-buffered LNPs displayed greater size heterogeneity (Figures S1a and S2). Despite comparable external morphologies, the transfection efficiency in HepG2 cells varied markedly, with fresh citrate-buffered mRNA-LNPs demonstrating the highest performance in all three studied ionizable lipids (Figure 1c). For instance, SM-102 mRNA-LNPs in citrate storage buffers exhibited 151% higher transfection efficiency compared to the same nanoparticles in Tris storage buffers. Therefore, it is necessary to investigate the internal morphology to understand these profound differences.

### pH-Dependent Phase Transitions of Empty LNPs

LNPs are internalized through multiple endocytic pathways, including macropinocytosis, clathrin-mediated endocytosis and caveolae-mediated endocytosis.^39^ To achieve efficient functional delivery, the mRNA payload must escape into the cytosol before late endosomes mature into lysosomes, thereby avoiding enzymatic degradation.^39^ LNPs encounter pH values of 6.0–6.5 in early endosomes and 5.0–5.5 in late endosomes.^40^ Such pH transitions change the charged state of ionizable lipids (p*K*_a_ ≈ 6–6.7), which often produce phase transitions in the LNP internal structure that are linked to endosomal escape efficiency.^12,21,41^

To better understand the phase behavior along the endosomal pathway, we examined the internal phase of empty LNPs (non-mRNA loaded) across different pHs of 4.5–7.4 (Figure 2) to provide a foundation for later examining more complex mRNA-LNPs. The *d*-spacing between the ionizable lipid aqueous domains is derived from SAXS (Figure S3) based on Bragg’s Law, which reflects the repeated separation between scatterers. Depending on the internal ordering, some samples exhibited a single broad peak, while others showed multiple peaks. The ratio of q-values of well-resolved peaks can be used to identify the ordered phase. For example, a peak ratio of 1:√3:2:√7 signifies an H_II_ phase. We find that at pH 7.4 with non-protonated ionizable lipids, a disordered structure without a peak was present. By decreasing the pH to 6.5, an inverse micellar (L_II_) phase appears, typically with one broad peak, consistent with previous findings.^12,26^ Further decreasing the pH to 5.5 or 4.5 caused the formation of an H_II_ phase. Across all studied ionizable lipids, these transitions from L_II_ to an H_II_ take place sooner (*i.e.*, at higher pH values) for citrate-buffered LNPs relative to the other buffers (Figure S4). These citrate-based samples also have more defined diffraction peaks in lower pH conditions, signifying stronger and more consistent ordering. An H_II_ phase transition earlier in the acidification process promotes earlier endosomal escape,^12,26^ which is likely the reason for the increased transfection efficiency shown in Figure 1. Citrate has previously been shown to facilitate an earlier onset of the H_II_ phase compared to phosphate and acetate buffers for MC3 mRNA-LNPs, which was correlated with enhanced protein expression.^26^

**Figure 2:**
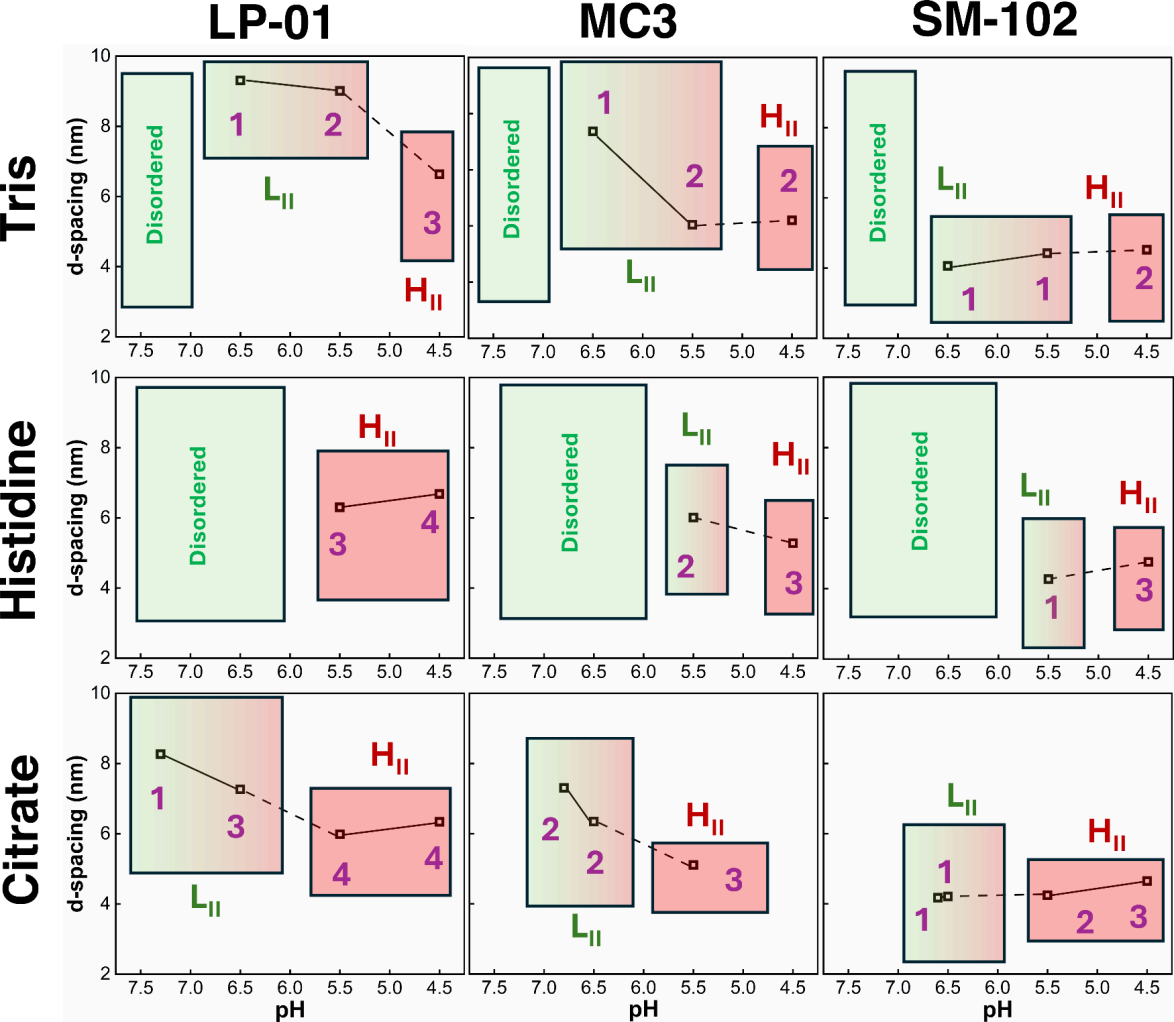
Effect of buffer species and ionizable lipid on empty LNP internal structure. Empty LNPs with LP-01, MC3 and SM-102 ionizable lipids in Tris, histidine and citrate storage buffers were studied at different pHs, where the phase change is shown by the change from green to pink shading. Using SAXS profiles in Figure S3, *d*-spacing was calculated from Bragg’s law. Near each *d*-spacing point, the number of well-defined diffraction peaks is added in purple.

The transition from L_II_ to H_II_ upon acidification is driven by protonation of ionizable lipid headgroups, which increases their effective area and promotes less negative mean curvature, favoring the H_II_ phase.^12,42,43^ This phase change is shown by the change from green to pink shading in Figure 2. The *d*-spacing between cylinders in the H_II_ phase is the lowest observed (4–6.5 nm), as expected given the smaller H_II_ cylinder radius versus L_II_ spheroidal radius.^12^ Within the H_II_ phase region, further decreasing the pH caused a slight increase in the *d*-spacing, likely as a result of increased charge–charge repulsion between ordered units with the protonated head groups of ordered ionizable lipids. Among examined ionizable lipids, the *d*-spacing is smallest for the empty LNPs with SM-102, perhaps due to their lower water content.^44^ Interestingly, SAXS for LP-01 with citrate buffer shows four H_II_ diffraction peaks, the highest number seen, and transitions at the earliest pH (Figure 2 and Figures S3 and S4). For all ionizable lipids, the earliest H_II_ phase transition and strongest ordering for citrate buffer samples (Figure 2) correspond to the highest transfection efficiency (Figure 1), consistent with fusogeneis of excess noncomplexed lipid arrays with endosomal membranes.

### Internal Structure and Cellular Properties of mRNA-Loaded LNPs

At our highest pH (7.4 for Tris and histidine, 6.9 for citrate) the incorporation of mRNA produced a weak SAXS diffraction peak (q ≈ 0.085–0.15 Å^–1^ in Figure 3 and Figure S3), attributed to weak ordering of mRNA-ionizable lipid complexes, as has been seen previously.^21,37,45,46^ Given that the peaks were asymmetric, they were deconvoluted into two peaks representing separate phases, along similar lines as in recent studies.^19,21,46,47^ The two deconvoluted phases represent 1) a mRNA-lipid complex peak and 2) a peak from excess lipid ordered domains with a much lower mRNA content, as shown in Figure 3, Figure S5 and Tables S1 and S2.^47,48^ In LP-01 and MC3 samples, the mRNA-lipid peak occurs at a higher q than the excess lipid peak, but the order is switched for SM-102 LNPs (Figure S5). This switch is a result of a difference in the location of excess lipid packing peaks for different ionizable lipids, while the mRNA-lipid peaks are at fairly consistent spacing for all ionizable lipids (Figure S5). The location of the excess lipid peak is consistent with the corresponding empty LNP peaks and prior literature for MC3 and SM-102 lipids.^47,49^ To evaluate how mRNA-lipid interactions influence internal ordering, 50 mM Tris mRNA-LNP at varying N/P ratios (1, 3, 4.5 (original), 9, and 12) were analyzed by SAXS (Figure S6). Increasing the N/P ratio (corresponding to lower mRNA loading) led to an increase in the peak area associated with excess ionizable lipid, accompanied by a decrease in the mRNA-lipid-associated peak, corroborating the peak deconvolution analysis.

**Figure 3:**
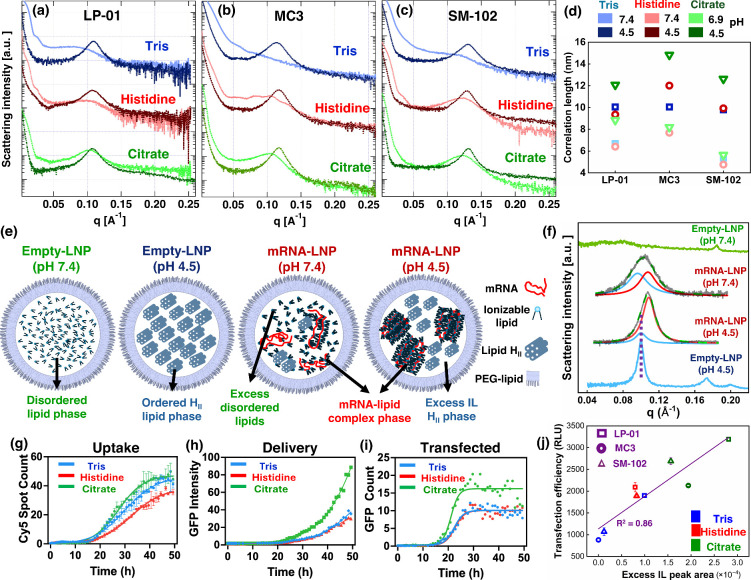
pH-dependent ordering of FLuc mRNA-LNPs and its correlation with *in vitro* studies. (a–c) SAXS profiles of mRNA-containing LNPs at pH 7.4 (light color) and at pH 4.5 (dark color) with (a) LP-01, (b) MC3 and (c) SM-102 ionizable lipids in different storage buffers. The pH color map in panel (d) corresponds to all data in panels (a–d). The citrate-buffered sample reached a pH of 6.9, as further adjustment to pH 7.4 was not achievable. (d) Correlation lengths of the primary mRNA-lipid peaks, calculated from fitting the SAXS data in (a–c). (e) Schematic illustrations of empty and mRNA-loaded LNPs at pHs of 7.4 and 4.5, together with the SAXS schematic of corresponding structures. The empty LNP at pH 7.4 shows a disordered internal structure with no noticeable scattering peak. Upon decreasing the pH to 4.5 the ionizable lipids form an inverse hexagonal phase with sharply defined ordering peaks in SAXS. For mRNA-LNPs, two ordered phases coexist: mRNA-lipid complexes and excess ionizable lipids, with two overlapping SAXS peaks that can be deconvoluted. By decreasing the pH, the mRNA-lipid complex phase becomes more pronounced and almost all the excess lipid region forms the H_II_ phase. (f) An example of peak deconvolution of LP-01 citrate-buffered mRNA-LNPs at pHs of 7.4 and 4.5, together with their empty LNPs. The red and blue dashed lines are peak fits for mRNA-lipid and excess lipid phases, respectively, and the dashed green line is the cumulative fit. Fluorophore-labeled Cy5-GFP mRNA-LNP with LP-01 ionizable lipid in studied storage buffers: (g) cellular uptake quantified as Cy5 puncta per nucleus, (h) GFP mRNA-LNP delivery quantified as mean GFP fluorescence intensity per nucleus, and (i) transfected GFP count quantified by the number of GFP-positive cells, representing successful mRNA delivery and expression. (j) Correlation between FLuc transfection efficiency and SAXS excess ionizable lipid peak area at neutral pH across all buffers and ionizable lipids.

At pH 7.4, there is a weak interaction between partially protonated ionizable lipids and anionic mRNA cargo, as a result of an increased ionizable lipid p*K*a in the presence of RNA, forming the mRNA-lipid complex. It has been shown that the negative electrostatic potential of mRNA can modulate the protonation state of ionizable lipids, increasing the apparent p*K*a of lipids in close proximity to the mRNA,^47,50,51^ where some excess ionizable lipids near mRNA can form an ordered phase. Upon decreasing the pH to 4.5, the peaks became much more intense and narrow. The *d*-spacing of the higher-q peak at q ≈ 0.1–0.15 Å^–1^ decreases as the more protonated IL interacts more strongly with the mRNA. A key realization is that the excess lipid peaks of mRNA-LNPs align with the primary peaks of the corresponding empty LNPs at pH 4.5 (Figure 3f and Figure S5). At this pH, almost all of the excess ionizable lipids form an H_II_ phase without mRNA present (Figure 2 and Figure S5). However, the strong scattering contribution from the mRNA-lipid peak, shown in Figure S7, masks the region where the higher-order H_II_ peaks would appear. Figure 3d and Table S1 show that decreasing the pH leads to a higher correlation length and smaller *d*-spacing in the primary peak, owing to the stronger interaction between mRNA and more protonated ionizable lipids, as seen previously.^47^

An H_II_ phase is not expected for mRNA-rich domains as the secondary structure of mRNA perturbs long-range ordering of ionizable lipids in the LNP core relative to more linear RNA structures such as poly­(A) or siRNA.^12,37^ However, the excess cationic lipid domains that coexist with the mRNA-lipid complex phase can still exhibit the L_II_ to H_II_ phase transition similar to the empty LNPs, which contributes to fusogenic activity and mRNA endosomal release.^12,48^ In all buffers and ionizable lipids, the correlation length increases with pH to sufficiently high values to indicate ordering in both mRNA-lipid and excess lipid phases (Figure 3d and Tables S1 and S2). Moreover, the correlation lengths are higher for citrate buffers, as were the peak intensities. A schematic illustration of empty and mRNA-loaded LNPs at pHs of 7.4 and 4.5 is presented in Figure 3e together with an example of their scattering profiles in Figure 3f, highlighting the coexisting structure of mRNA-lipid and excess lipid phase domains.

To further understand the impact of LNP storage buffer on each step of RNA delivery, we used LNPs formulated with a 1:1 wt % ratio of GFP mRNA and a fluorescent, Cy5-labeled GFP mRNA to track cellular uptake (Cy5 fluorescence) and transfection efficiency (GFP expression) separately (Figure 3g–i). LNPs were formulated and buffer exchanged in an identical manner to the results above for FLuc mRNA-LNPs, and for this experiment, were incubated with HeLa cells. We observed that while buffer identity affected cellular uptake of RNA-LNPs to some degree, the buffer impacted GFP transfection to a much greater extent, with fresh citrate-buffered mRNA-LNPs again demonstrating the highest transfection efficiency. Collectively, these results suggest that the potency advantage of citrate arises predominantly at the endosomal escape stage, consistent with the earlier onset of H_II_ phase formation observed by SAXS.

Excess ionizable lipids exhibit greater molecular mobility than tightly packed mRNA-lipid complexes, since the larger complexes produce steric crowding that restricts lipid motion.^12,48,52^ This has been previously observed in temperature-dependent NMR studies in which ionizable lipids were shown to be more rigid and less dynamic in LNPs loaded with siRNA versus empty LNPs.^53^ It was proposed that two distinct populations were present with varied mobility, as seen in our SAXS data. As the pH decreases in endosomes, we expect these excess ionizable lipid domains with H_II_ structure may migrate more rapidly toward the LNP surface and promote fusion with endosomal membranes to allow the complex to escape and release the mRNA for translation.^48,54^ To better quantify this behavior, we compare the FLuc mRNA transfection efficiency with the excess ionizable lipid peak area across all lipid types and storage buffers from the data in Figures 1c and 3a–c and Figure S5 to show a strong correlation between these two parameters (Figure 3j). While the citrate samples were at a slightly lower pH, we do not expect this to result in a significant change in the correlation because we find that the excess ionizable lipid peak area undergoes minimal change with pH in the samples tested (Figure S8a). In order to capture the effect of peak sharpness, we define an ordering parameter as the peak intensity/width (eq S2), and find similar success for correlating the transfection efficiency (Figures S8 and S9). Overall, these findings indicate that the choice of buffer species is a critical design parameter to modulate the initial CQAs and internal structure of mRNA-LNPs. The formation of favorable internal structures (*i.e.*, H_II_ phase) earlier in the endosomal acidification process significantly enhances transfection efficiency while preserving appropriate size, internal morphology and encapsulation efficiency.

### Freeze–Thaw Stability

Long-term storage of mRNA-LNPs commonly utilizes freezing at various temperatures due to the fragility of the encapsulated mRNA cargo. While this method of storage mitigates mRNA degradation (*e.g.,* hydrolysis), it poses challenges in maintaining the structural integrity of the drug product after a freeze–thaw cycle. The glass transition temperature of the maximally freeze-concentrated phase (*T*_g_’) was measured in the range of −35 to −40 °C for the studied formulations (Figure S10). In the interest of staying well below the *T*_g_’ and ensuring a rigid glassy matrix with reduced mobility,^55^ we proceeded with freezing mRNA-LNPs at −80 °C. Moving forward, we focused on the FLuc mRNA-LNPs with top performing LP-01 lipids for all three storage buffers. Figure 4 shows that the average hydrodynamic diameter of mRNA-LNPs in Tris and histidine increased by a modest 24% and 51%, respectively, while in citrate buffer the size increased by over 918%, following one cycle of freezing at −80 °C and thawing at 5 °C. This pronounced aggregation led to a complete loss of both encapsulation and transfection efficiency (Figure 4b,c). These losses in both categories were much more moderate for the histidine-buffered samples, while Tris stood out with minimal loss of either encapsulation or transfection efficiency (Figure 4). To assess the generalizability of these buffer-dependent potency trends across cell types, we extended transfection studies from HepG2 to HEK293T and Jurkat cells (Figure 4c), observing consistent behavior across all three human cell lines (note that citrate-buffered formulations achieved the highest transfection efficiency prior to freezing, consistent with earlier results (Figure 3)). SAXS profiles (Figure 4d) further revealed that the freeze–thaw cycle induced structural changes across all buffer conditions relative to fresh formulations. The ordering peak intensity decreased, while the low-q region indicated morphological transitions from spherical to anisotropic particles. Notably, LNPs in citrate lost any distinct nanoparticle scattering profile, consistent with extensive aggregation, where a noticeable drop in low-q intensity is observed, and the ordering peak at high-q region disappeared (Figure S11). These findings may be consistent with those of Cheng et al.,^24^ who observed that citrate buffer is the most fusogenic buffer compared to phosphate and acetate buffer, where only 25 mM citrate concentration caused fusion of vesicles at pH 4. Such fusion can occur during freezing, as ice crystallization leads to solute cryoconcentration and localized pH shifts for citrate-containing buffers to below 5.9.^56^ The −3 charged citrate may cause multivalent ion bridging of the small amount of positively charged ionizable lipids on the surface of the LNPs. Even minor pH decreases can enhance protonation of ionizable lipids, altering their charge state and promoting lipid rearrangement, fusion, or leakage of encapsulated mRNA.

**Figure 4:**
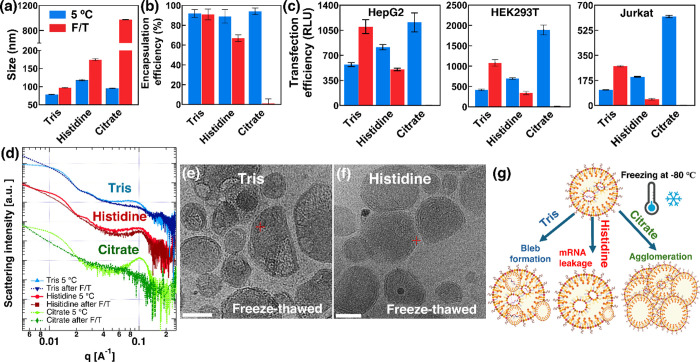
Freeze–thaw stability of mRNA-LNPs in different storage buffers at 50 mM. (a) Size, measured with DLS (b) encapsulation efficiency, determined by RiboGreen assay and (c) transfection efficiency of mRNA-LNPs formulated in Tris, histidine, and citrate buffers in HepG2, HEK293T and Jurkat cell lines was evaluated using the Bright-Glo Luciferase Assay. Measurements were performed before freeze–thaw (stored at 5 °C, blue) and after freeze–thaw (stored at −80 °C, red). (d) SAXS profile of mRNA-LNPs before (5 °C) and after freeze–thaw (F/T) in Tris, histidine and citrate buffers. Cryo-TEM images of mRNA-LNPs in (e) Tris and (f) histidine buffers after freeze–thaw. The pH is 7.4 for Tris and histidine, and 6.9 for citrate. Scale bars = 60 nm. (g) Schematic of freeze–thaw stability of mRNA-LNPs with different storage buffers.

Cryo-TEM imaging confirmed bleb formation in 50 mM Tris-buffered LNPs after freeze–thaw (Figure 4e), likely arising from stress-induced lipid and mRNA rearrangements leading to phase separation. Blebs can be either empty or mRNA-loaded, where the former likely arises from mechanical stresses introduced during formulation, lyophilization, or freeze–thaw treatment.^28,46,57^ Similarly, Tris-buffered mRNA-LNP exhibits both mRNA-containing (mottled contrast in TEM) and empty blebs (less contrast) after the freeze–thaw cycle, which the former can be beneficial for transfection efficiency. The structural perturbations in Tris-buffered LNPs induced by bleb formation, together with the retention of encapsulation efficiency (mRNA-loaded blebs), contributed to enhanced transfection potency following freeze–thaw cycling. Henderson et al.^28^ similarly reported that Tris buffer enhanced freeze–thaw stability relative to PBS at −20 °C, resulting in improved *in vivo* transfection. This stabilizing effect arises from several physicochemical properties of Tris: (1) it has a relatively high buffer capacity around pH 7–9 and a lower tendency to crystallize during freezing, (2) Tris hydroxymethyl groups can form hydrogen bonds with water molecules, reducing ice nucleation and growth,^35,58^ and (3) Tris can function as an effective aldehyde scavenger, reacting with lipid-derived oxidative impurities that would otherwise result in mRNA-lipid adducts, leading to retained drug product potency.^35,59–61^ Figure 4g shows a schematic summarizing these findings. Particularly notable features with Tris-, histidine-, and citrate-buffered mRNA-LNPs were bleb formation, mRNA leakage, and aggregation, respectively, after the freeze–thaw process. Together, these effects can suppress particle aggregation, reduce osmotic and mechanical stresses during freezing, and maintain a more stable protonation state of the ionizable lipid. Consequently, Tris acts not only as a buffer but also as a cryoprotectant, helping LNPs retain both structure and biological function after freeze–thaw.

To further understand the impact of buffer for long-term storage, we compared mRNA-LNPs stored at 5 °C and frozen at −80 °C for one month (Figures S11 and S12). After one month at 5 °C, the LNP sizes only changed for the histidine-buffered LNPs from 117 to 190 nm (note this size increase was unexpected given our previous findings on histidine-stabilized LNPs,^22^ though this formulation notably differs in lipid, cargo, buffer strength, pH, salt and sucrose content). Notably, transfection efficiency significantly decreased for histidine and citrate samples, but was stable for Tris-buffered samples at 5 °C. After one month at −80 °C, then thawed at 5 °C, all properties remained fairly constant in Tris buffer, but transfection efficiency dropped markedly in histidine and citrate. Even for freeze–thaw without one month storage, the mRNA encapsulation was greatly diminished in citrate samples, perhaps as a consequence of aggregation and large structural changes. SAXS profiles (Figure S11) further corroborate the superior stability of Tris-buffered LNPs at both storage conditions, exhibiting small changes in scattering curves before and after storage. For one-month storage, Tris-buffered LNPs at −80 °C provided sufficient stability with respect to encapsulation efficiency and transfection potency. Increasing the sucrose content within these Tris-based formulations from 5 to 10 w/v% did not result in major structural differences by SAXS (Figure S13). These results suggest that increasing sucrose concentration may not significantly perturb the internal organization of LNP components, although this could be formulation-dependent.

### Impact of Tris Concentration

Buffer concentration is a critical parameter in controlling the internal structure and CQAs of mRNA-LNPs, primarily by modulating lipid–lipid, lipid-mRNA and LNP-LNP interparticle interactions.^24,27^ Given that Tris buffer provided superior stabilization during freeze–thaw cycles and one-month storage at the 50 mM concentration above, it was also studied at 10 and 150 mM. Interestingly, while 10 mM Tris showed an increase in particle size and a slight decrease in encapsulation efficiency upon freeze–thaw, formulations with 50 and 150 mM Tris exhibited only moderate size changes (Figure 5a and Figure S14a), suggesting that 10 mM Tris is not sufficient to maintain structural stability in this formulation. Consistent with this, freeze–thawed FLuc mRNA-LNPs formulated at 50 and 150 mM Tris showed a higher transfection efficiency compared to 10 mM in all three studied cell lines (Figure 5b). mRNA-LNPs in 50 and 150 mM Tris yielded comparable transfection levels in HepG2 and HEK293T cells, whereas Jurkat cells exhibited a slightly higher transfection efficiency at 150 mM Tris (Figure 5b). Similarly, the cellular trafficking assays revealed that the cellular uptake and delivery efficiencies of fresh GFP mRNA-LNPs in 50 and 150 mM Tris buffers outcompeted those stored in 10 mM Tris (Figure 5c,d and Figure S14b). The reduced uptake at 10 mM Tris suggests that insufficient buffering capacity compromises particle properties that govern cellular internalization, rather than endosomal escape and intracellular cargo release.

**Figure 5:**
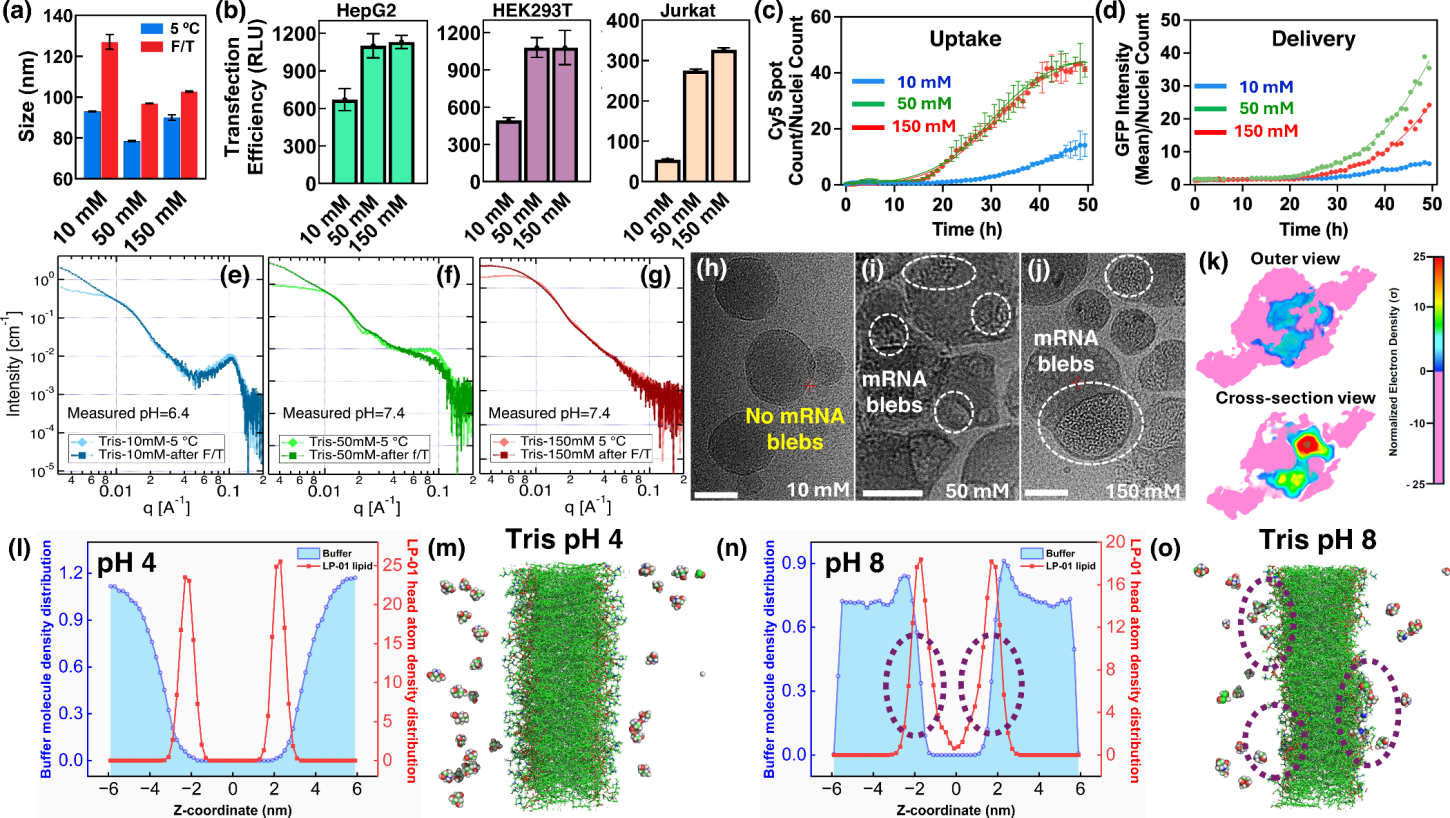
Effect of Tris molarity on LP-01 mRNA-LNPs structure, freeze–thaw stability and cellular delivery. (a) Size obtained from DLS at fresh (5 °C) and after freeze–thaw (F/T), and (b) transfection efficiency in 10, 50, and 150 mM Tris buffer after freeze–thaw in HepG2, HEK293T and Jurkat cell lines. (c) Cellular uptake quantified as Cy5 puncta per nucleus, and (d) delivery quantified as mean GFP fluorescence intensity per nucleus for fresh LP-01 mRNA-LNPs in Tris storage buffer with different Tris molarities. Freeze–thaw stability of mRNA-LNPs with different Tris molarities: (e) 10 mM, (f) 50 mM, and (g) 150 mM, revealed by SAXS. 5 °C samples are before freeze–thaw. Cryo-TEM of mRNA-LNPs in (h) 10 mM, (i) 50 mM, and (j) 150 mM Tris after freeze–thaw. The measured pH for 50 mM and 150 mM Tris is 7.4, while for 10 mM is 6.5. Scale bars = 60 nm. (k) DENSS relative electron density distributions for fresh 150 mM Tris showing electron dense RNA regions and lipid regions (pink) with negative density relative to the solvent. MD simulations on LP-01-cholesterol-DSPC in Tris buffer, at pH 4 (l, m) and pH 8 (n, o). At pH 8, there are strong interactions of buffer molecules with the LP-01 ionizable lipid head atom with storage buffer molecules, highlighted with dashed circles.

In addition to the above CQAs, modifying Tris molarity produced a profound effect on mRNA-LNP internal structure both upon formulation and after one freeze–thaw cycle (Figure 5e–g and Figure S14). At only 10 mM Tris, a pronounced SAXS peak around 0.106 Å^–1^ for mRNA-LNPs indicated an ordered structure (Figure 5e). Here, the buffer capacity was insufficient to maintain pH 7.4, resulting in a pH of 6.5, which facilitated the formation of an ordered internal structure with a *d*-spacing of 5.9 nm. The intensity of this ordering peak progressively diminished and disappeared with increasing Tris molarity to 50 and then 150 mM, while the pH raised to 7.4 (Figure 5e–g). Similar behavior was observed for mRNA-LNPs with MC3 and SM-102 lipids (Figure S15). Increasing the Tris concentration raises the ionic strength of the solution, as it is partially protonated at physiological pH. To isolate the effect of ionic strength, we buffer exchanged mRNA-LNPs into 10 and 50 mM Tris formulations with elevated sodium chloride concentrations that match the ionic strength of the 150 mM Tris formulation (Figure S16). From SAXS data, the ordering was independent of ionic strength at a given Tris content, but varied with Tris content at a given ionic strength, demonstrating that ordering was controlled predominantly by buffer concentration and not simply ionic strength.

The SAXS profiles for mRNA-LNPs in 150 mM Tris underwent minimal if not negligible changes before and after freeze–thaw (Figure 5g), indicating preservation of internal structure. In contrast, the peak intensity dropped modestly at 50 mM Tris. Cryo-TEM images revealed that increasing Tris concentration leads to bleb formation (Figure 5h–j and Figure S17). While LNPs in 10 mM Tris after freeze–thaw showed no mRNA-rich blebs (Figure 5h and Figure S17), as Tris molarity increased, the prevalence of mRNA-containing blebs with mottled contrast also increased, with freeze–thawed 150 mM Tris LNPs exhibiting the highest number (additional cryo-TEM images are provided in Figure S17). The density from solution scattering (DENSS)^62,63^ reconstruction of the fresh 150 mM Tris mRNA-LNP sample reveals an anisotropic electron density distribution. The negative contrast scattering correlates with lipid components with less scattering than the solvent and can show the overall morphology of the particle.^62^ The positive electron density region, corresponding to mRNA-rich regions, which possesses a higher scattering length density than the surrounding lipids,^64^ appears to be localized within a single domain, suggesting the formation of an mRNA-loaded bleb-containing structure (Figure 5k). It should be noted that SAXS measures ensemble-averaged scattering from the bulk mRNA-LNP population, and hence, the DENSS reconstructions reflect average electron density distributions rather than individual particle structures. Consequently, heterogeneity in size or internal organization may influence the observed features, and the models represent the dominant population average.^65^ Moreover, the nonspherical shape of a bleb-containing LNP is further indicated by an asymmetrical biomodal distribution function P­(r) (Figure S14d,e). The minimum accessible scattering vector (*q*_min_ = 0.004 Å^–1^) provides adequate low-q coverage to capture the Guinier regime and reliably determine overall particle dimension (*D*_max_), supporting meaningful P­(r) analysis. Although the sample exhibits structural heterogeneity, ensemble averaging in SAXS typically broadens P­(r) features rather than generating artificial bimodality.^66^

The mRNA-rich blebs in the fresh 150 mM Tris sample were mostly preserved upon freeze–thaw, with almost no empty blebs, as observed in cryo-TEM (Figure 5j). Although the role of blebs as a positive or negative attribute is not fully understood,^67^ there are reports in which an increased number of mRNA-filled blebs can enhance transfection potency.^24,67^ It has been hypothesized that RNA localized in blebs may be protected by limiting exposure to ionizable lipids, which can otherwise form adducts with mRNA.^24,68,69^ For our samples, RNA integrity analysis by high-performance liquid chromatography (HPLC) demonstrated that mRNA-LNPs in 10 mM Tris exhibited noticeable mRNA-lipid adducts after one month storage at 5 and −80 °C, which was mitigated with increasing Tris content to 50 and 150 mM (Figure S18). This reduction in RNA-lipid adducts may either be due to increased bleb formation or Tris acting as an aldehyde scavenger.^61^

The interplay of buffer, mRNA and lipid interactions is critical to understand LNP morphology and bleb formation. For instance, Cheng et al.^24^ proposed that increasing sodium citrate buffer molarity during formulation would produce mRNA-loaded particles with empty blebs at acidic pH; however, as LNPs are brought to physiological pH, the deprotonation of ionizable lipids weakens interactions with mRNA and subsequently drives mRNA into the blebs. Here, MD simulations (Figure 5l–o and Figures S19–S21) indicate Tris buffer forms hydrogen bonds with the LP-01 ionizable lipid at high pH (Figure S20), whereas such hydrogen bonds are not detectable at low pH. At high pH, Tris forms approximately twice as many hydrogen bonds with LP-01 as histidine, whereas hydrogen bonds are not detectable for citrate (Figure S21). The density distribution profiles for Tris show prevalent spatial overlap between the LP-01 headgroup nitrogen atoms and the buffer molecules at pH 8 (Figure 5n,o) for Tris, but much less so at pH 4 (Figure 5l,m) or for the other studied buffers (Figure S19). This hydrogen-bond formation by Tris stabilizes the local environment of the LP-01 headgroup in its neutral form and weakens competing intermolecular interactions. Furthermore, at high pH the partially protonated form (Tris^+^) can penetrate the lipid surface (Figure 5n,o), which can compete with partially protonated LP-01 for electrostatic interactions with negatively charged mRNA. MD simulations show that increasing the Tris molarity to 150 mM further raises the number of hydrogen bonds with the ionizable lipid (Figures S22 and S23) and further weakens the competing mRNA-lipid interaction, promoting mRNA dissociation and bleb formation at high pH. In contrast, increasing the histidine buffer molarity to 150 mM at pH 7.4 did not lead to noticeable external or internal structural changes or CQA changes (Figure S24), either prior to or following freeze–thaw. This lack of sensitivity can be attributed to the weaker hydrogen-bonding interaction of the near-neutral histidine buffer with LP-01 at high pH (Figure S19), which is approximately half that of Tris (Figure S21). For citrate, MD simulation results at pH 4 show noticeable penetration of anionic citrate buffer molecule that forms hydrogen bonds with LP-01 and electrostatic attraction with the cationic ionizable lipids, bringing them into close proximity (Figures S19–S21). This enhanced interaction suggests that citrate more effectively stabilizes the fully protonated lipid state and promotes H_II_ phase, which may underlie its superior prefreeze performance. This behavior is consistent with prior reports^26^ showing that buffer species can modulate ionizable lipid properties by altering the effective headgroup area and charge. Specifically, negatively charged citrate associates with protonated amine groups, reducing electrostatic repulsion and increasing the bending rigidity, effects that favor H_II_ phase stabilization at higher pH. These simulation results suggest that buffer molecules can reside inside the LNP and be in contact with ionizable lipids through hydrogen bond formation, leading to a permanent buffer impact on the internal structure and consequently the transfection efficiency. These data corroborate why even after dilution in cell media for *in vitro* studies, the impact of storage buffer persists.

The combination of bleb formation and preserved scattering features suggests that structure contributes to improved freeze–thaw stability of mRNA-LNPs at 50 and 150 mM Tris. Regarding the stability, the mRNA length and its secondary/tertiary structure play important roles in the stability of mRNA-LNP products.^35^ The pH-dependent mRNA cleavage model^70^ indicates that mRNA has a longer half-life in aqueous solution than inside LNPs. Proton partitioning in the lipid phase elevates the local internal pH, which in turn accelerates base-mediated backbone cleavage.^35,71,72^ When mRNA is encapsulated in aqueous bleb pockets of the LNP, such interactions that disturb RNA integrity may be decreased, contributing to the enhanced freeze–thaw stability of bleb-containing LNPs. The comparison of the pH-dependent structural ordering behavior of fresh 50 mM (no blebs) and 150 mM (bleb-containing) Tris mRNA-LNPs shows that the bleb-containing morphology possesses a higher excess lipid phase ordering parameter at pH 6.5–4.5 due to more available ionizable lipids that do not interact with the bleb-containing mRNA (Figures S25–S27). Increased ionizable lipid availability would promote formation of a more pronounced H_II_ phase, which could contribute to the enhanced transfection efficiency observed for bleb-containing mRNA-LNPs (Figure S28). Cryo-TEM shows straight lines and hexagons indicative of an H_II_ phase, which we expect are in the excess ionizable lipid regions (Figure S25f). The peak area corresponding to the excess ionizable lipid phase, and its ratio relative to the mRNA-lipid phase, decreases with acidification in the nonbleb 50 mM fresh Tris formulation, likely due to increased protonation and stronger lipid-mRNA association. In contrast, the bleb-containing freeze–thawed 50 and 150 mM Tris formulations maintain similar peak areas and phase ratios upon acidification, suggesting preservation of excess ionizable lipid domains due to RNA entrapment in blebs. Therefore, we believe that in addition to reducing mRNA-lipid adduct formation, the preservation of excess lipid phases likely promotes endosomal escape, leading to increased transfection efficiency with blebs. Figure S28e provides a schematic mechanistic interpretation of this behavior.

Even with these findings, isolating the specific contribution of bleb morphology to transfection efficiency is admittedly challenging, as bleb formation is inherently coupled to other formulation variables. Still, our data provide convergent evidence linking blebs to enhanced potency through a defined structural mechanism. First, we observe a strong correlation between excess ionizable lipid peak area and transfection efficiency across all lipid types and buffers (Figure 3j). Second, cellular trafficking assays demonstrate that potency differences between citrate, histidine and Tris formulations arise predominantly at the RNA delivery and transfection stages, rather than at cellular uptake (Figure 3g–i). Third, pH-dependent SAXS deconvolution shows that bleb-containing formulations (*e.g.*, freeze–thawed 50 and 150 mM Tris) maintain a higher extent of excess ionizable lipid across the endosomal pH range (Figure S28), consistent with greater availability of ionizable lipids for H_II_ phase formation. Together, these findings support a mechanistic model in which bleb formation promotes segregation of mRNA into aqueous compartments, increasing the fraction of excess ionizable lipid available for fusogenic H_II_ phase transitions during endosomal acidification. While direct causal isolation of bleb-mediated endosomal escape requires further study, the present work identifies relationships between storage buffer identity, bleb morphology, and intracellular delivery efficiency. These findings highlight that Tris molarity is a critical parameter for fine-tuning LNP physicochemical properties, providing a simple yet effective strategy to maximize both stability and functional potency.

To examine how subtle changes in buffer protonation state affect LNP structure and function, the pH of 50 mM Tris-buffered mRNA-LNPs was increased from 7.4 to 8: the p*K*a of Tris (Figure S29). This change to pH 8 resulted in a moderate increase in transfection efficiency for the fresh formulation but a significant preservation of size and transfection efficiency after a freeze–thaw cycle. The peak in the scattering profile at 5 °C decayed at pH 7.4 after freeze–thaw. In contrast, in both cases, a peak was not present at pH 8 and the scattering profile did not change with freeze–thaw (Figure S29d,e). Complementary cryo-TEM imaging revealed extensive bleb formation in fresh mRNA-LNPs at pH 8, closely resembling the morphology observed at a higher concentration of 150 mM Tris molarity at pH 7.4 (Figure 5j). These significant changes are remarkable given such a small change in pH. The likely reason is that ionizable lipids would have different p*K*a based on their locations in the LNP as those interacting with mRNA possess higher p*K*a values,^51^ and mRNA is believed to prevent full deprotonation of ionizable lipids.^50^ In this regard, increasing the pH of the Tris buffer to 8 can cause further deprotonation of ionizable lipids that can lead to a loss of their attraction with mRNA, resulting in the separation of mRNA molecules into blebs.

## Conclusions

This comprehensive mechanistic study reveals that buffer identity and concentration critically govern the size, internal organization, stability, and transfection efficiency of mRNA-LNPs before and after freeze–thaw storage. For both empty and mRNA-LNPs prior to freeze–thaw, we observe that citrate buffers promote an earlier pH transition to an inverse hexagonal (H_II_) phase compared to Tris and histidine buffers. Interestingly, citrate formulations also exhibit the highest transfection efficiency of FLuc and GFP mRNA cargoes in four diverse cell lines (HepG2, HeLa, HEK293T and Jurkat), corroborating that the H_II_ phase favors fusogenesis with endosomes. As seen previously,^12^ internal LNP structures are perturbed by encapsulated mRNA cargo,^12,37^ thus lacking the definitive SAXS peaks associated with H_II_, inverse micellar (L_II_) and inverse lamellar (L_α_) phases that are effectively resolved in empty LNPs. To address this limitation, we resolve two SAXS peaks associated with mRNA-lipid stacks and mRNA-lean excess ionizable lipid phases with low mRNA content. This phase identification was further supported by mRNA-LNPs with different N/P ratios, where by increasing the N/P ratio (lower mRNA loading), a higher excess ionizable lipid peak was observed. On the basis of a recent simulation study^48^ on ionizable lipid mobility, we propose that the greater mobility of the excess ionizable lipids facilitates the formation of the fusogenic H_II_ phase that was present in the empty LNPs.

Freeze–thaw experiments of mRNA-LNPs stored in the lead buffers reveal that the internal structural integrity and transfection efficiency were best retained by Tris buffer, as aggregation occurred with trivalent citrate. Modifications of the Tris formulation, including increasing Tris concentration to 150 mM or slightly increasing pH to 8.0, both induced a profound structural shift to mRNA-loaded bleb morphologies as the mRNA-ionizable lipid interactions were weakened. These storage buffer modifications represent new design rules through which bleb content can be modified. Bleb formation by increasing the Tris concentration to 50 mM and higher was positively correlated with higher transfection efficiency after freeze–thaw. The blebs appear to stabilize the mRNA while requiring fewer ionizable lipid-RNA interactions. This consequently increases the amount of excess mobile ionizable lipids dissociated from mRNA that then form a favorable H_II_ phase. These findings are corroborated by SAXS and cryo-TEM at endosomal pH values, even after the stress of freezing.

Overall, these findings highlight the pivotal role of storage buffer composition, concentration, and pH in shaping the structure–function relationship of mRNA-LNPs. While suitable storage buffers have traditionally been identified through screening, this process is often iterative, time-consuming, and may yield limited mechanistic insight that can be leveraged for future drug products. Conversely, we anticipate that the mechanisms described here will accelerate buffer optimization by providing molecular-level understanding for their improved performance. While the design principles for refrigerated buffers may not ultimately translate to long-term storage of mRNA-LNPs due to the inherent sensitivity of the mRNA cargo, these design principles could be applied to other clinically validated, shelf-stable nucleic acids (*e.g.,* chemically modified siRNAs, antisense oligonucleotides, guide RNAs, or editing oligonucleotides). Additionally, we envision that these concepts may be more widely applicable to other nanoparticle systems leveraged in RNA delivery, although we expect that the structure–function relationships will vary from system to system. These mechanistic insights highlight the importance of storage buffer selection in formulation development and translation of RNA-LNP therapeutics.

## Materials and Methods

### Materials and Equipment

Chemical reagents were purchased from Avanti, Teknova, Corning, or ThermoFisher and used without purification unless noted otherwise. Commercially available ionizable lipid was sourced from WuXi Biologics. Firefly luciferase mRNA was sourced from TriLink Biotechnologies (lot WOTL104059) at a concentration of 2.33 mg/mL with a full-length of 1929 nucleotides and a proprietary sequence. Centrifuge and Eppendorf microcentrifuge tubes were purchased from Fisher Scientific. DI water was supplied via Milli Q Integral 15 system. PD-10 column purification was performed via Cytiva SephadexTM G-25 M columns (CAT# 17085101) and manufacturer gravity protocol. Bath sonication was performed using a Branson Bransonic CPXH Digital Bath 1800. All heating used a VWR Model 1224 water bath set to 40 °C. Masses for analytical measurements were taken on a Mettler Toledo XSR204 or XSE204 analytical balance. Mixing was performed with a commercially available off the shelf impingement jet mixer. All nanoparticles were stored in Corning 2 mL cryo vials (CAT# 430488). All buffers prepared using commercially available reagents. HepG2 cells, a human hepatocellular carcinoma cell line, were obtained from ATCC (Cat# HB-8065) and cultured in EMEM (2 mM l-glutamine, 1 mM sodium pyruvate, 1500 mg/L sodium bicarbonate; ATCC Cat# 30–2003) supplemented with 10% characterized fetal bovine serum (Cytiva Cat# SH30071.03) and 1% Pen/Strep 100X solution (Gibco Cat# 15140–122). Jurkat cells, a human T lymphocyte cell line, were obtained from Wei-Lun Chen at Eli Lilly & Company and cultured in RPMI-1640 (2 mM l-glutamine, 25 mM HEPES; Gibco Cat#22400–089) supplemented with 10% heat-inactivated fetal bovine serum (Cytiva Cat#SH30071.03HI) and 1% Pen/Strep 100X solution (Gibco Cat# 15140–122). HEK293T cells, a human embryonic kidney cell line, were obtained from Wei-Lun Chen at Eli Lilly & Company and cultured in DMEM (4.5 g/L d-glucose, 2 mM l-glutamine, 110 mg/L sodium pyruvate) supplemented with 10% heat-inactivated fetal bovine serum (Cytiva Cat#SH30071.03HI) and 1% Pen/Strep 100X solution (Gibco Cat# 15140–122). All media used for cell work was first filtered through a 0.22 μm PES sterile filtration membrane prior to use (Millipore Cat# S2GPU05RE).

### RNA-Lipid Nanoparticle Formation Procedure

Lipid nanoparticles were formulated with the following procedure. First, ionizable lipid, cholesterol (Avanti), DSPC (Avanti), and DMG-PEG-2000 (Avanti) were removed from −80 °C storage and allowed to come to room temperature. In addition, mFLuc was removed from −80 °C storage and allowed to come to room temperature. Lipids were then weighed out into glass scintillation vials. In an example formulation, Cholesterol (63.6 mg) was placed in a vial and 4240 μL of EtOH was added to make a 15 mg/mL stock solution. The sample was then placed in a heated water bath. This was repeated for DSPC (26.6 mg lipid, 1060 μL EtOH, 25 mg/mL) and DMG-PEG-2000 (18.8 mg lipid, 940 μL EtOH, 20 mg/mL). After approximately 10 min of heating, if any undissolved lipid remained, the solutions were then briefly sonicated. Ionizable lipid, provided as a liquid solution, was diluted to 30 mg/mL with an appropriate amount of ethanol. The solution was then mixed thoroughly without heating. Once all stock lipid solutions had been made, they were then combined in a 20 mL scintillation vial and EtOH added for a final concentration of 33.7 mM lipid and a molar ratio of 45:44:9:2 (Ionizable Lipid:Cholesterol:DSPC:DMG-PEG-2000). mRNA stock solution was made by diluting an appropriate amount of 2.33 mg/mL stock to 0.2775 mg/mL with 50 mM sodium acetate pH 4.5. Solution was mixed thoroughly, and mRNA concentration was measured on a Thermo Scientific NanoDrop One with a custom factor of 37.55. The lipid and mRNA working solutions were then loaded into the mixing system. Samples were generated with a total flow rate of 30 mL/min, 3:1 mRNA:lipid volume ratio, utilizing an impinging jet mixer. Nanoparticles, with a 4.5:1 ionizable lipid:mRNA molar ratio, were collected in a tared 500 mL PETG media bottle. The first 2 mL, and last 1 mL of each batch were not collected, and the final weight of the LNP solution was collected post mixing. Following mixing, samples were buffer exchanged via PD-10 desalting columns into respective buffers and manufacturer provided gravity protocol. In all instances, buffers were pH adjusted to the target pH prior to use in buffer exchange (including within acidification experiments, *e.g.,* 4.5) and the final LNP solution was not pH adjusted further. In addition to N/P ratio of 4.5, mRNA-LNPs with N/P ratios of 1, 3, 9, and 12 were generated by decreasing the mRNA loading. LNPs were stored at −80 °C and thawed at 5 °C for the freeze–thaw stability experiments, while fresh samples were stored at 5 °C shortly before characterization

### General Lipid Nanoparticle Analysis Procedure

#### Size Analysis

The LNP solution was diluted 50x in PBS 1X (10 μL LNPs in 490 μL PBS 1X) in a ZEN0040 disposable cuvette. Size was analyzed with a Malvern Zetasizer Nano dynamic light scattering instrument, with the measurement angle of 173°. Samples were measured with the liposome default setting (refractive index of 1.45, absorption 0.001) and dispersant set to PBS (refractive index of 1.33, viscosity of 0.8872 mPa s). Temperature was set to 25 °C with an equilibration time of 30 s. Each sample was measured in automatic attenuation and positioning mode, and data were analyzed using the cumulant method to obtain the intensity-weighted Z-average hydrodynamic diameter. The PDI derived from cumulant analysis was used to assess sample dispersity. Instrument default settings were used for all other settings.

#### RiboGreen Analysis

Two standard curves of RNA were prepared, one curve of RNA in 1× TE buffer pH 5 (Prepared from Invitrogen 20× TE buffer Cat# R11490), and one of RNA in 0.1% Triton X-100 in TE (Prepared from Sigma-Aldrich Triton X-100). To start, 20 μL of 0.2 mg/mL RNA was added to 980 μL of 1× TE or 0.1% TX and mixed well for a 4000 ng/mL standard. Following this, 250 μL of the 4000 ng/mL standard was taken and serially diluted 1:1 to a concentration of 8 ng/mL. A blank was also prepared for each standard curve. Following this, samples were prepared. First, 10 μL of LNP was added to 90 μL of 1× TE and mixed thoroughly to make Sample Dilution 1. Following this, 10 μL of Sample Dilution 1 was added to 990 μL of 1× TE or 0.1% Triton X-100 in TE and briefly vortexed. After samples were prepared, 100 μL of the standard curves and samples were pipetted in duplicate to a 96-well plate (Fisherbrand Cat# 12565501). Ribogreen dye (Invitrogen Cat# R11491) was diluted 200x in TE buffer, mixed well, and 100 μL of diluted dye was added to each well of the 96-well plate. Immediately following this, samples were measured using a TECAN Spark 10 M plate reader with 480 nm excitation wavelength, and 520 nm emission wavelength.

#### RNA Integrity Analysis

High-performance liquid chromatography (HPLC) was performed to measure RNA integrity of LNP samples. Agilent 1260 Infinity series HPLC was used, equipped with a variable wavelength detector set to 260 nm. A reverse phase gradient method was used for separation. Mobile phase A was 100 mM Hexylammonium Acetate in water (diluted from 2.0 M stock, Glen Research, Cat # 60–4210–57), and mobile phase B was 100 mM Hexylammonium Acetate in 60:40 Acetonitrile:Water (Acetonitrile from Fisher Chemical, Cat # A955–4). The gradient starts with an isocratic portion at 10% mobile phase B for the first minute, then uses a succession of linear gradients to elute the RNA of interest. The gradients are as follows: from 1 to 3 min, mobile phase B increases from 10% to 60%; from 3 to 23 min, mobile phase B increases from 60% to 80%; from 23 to 23.5 min, mobile phase B increases from 80% to 100%; and from 23.5 to 28 min, mobile phase B decreases from 100% to 10%. It then stays isocratic until the 30 min mark. Flow rate was constant, at 0.4 mL/min. The column used was DNAPac RP, 4 μm, 2.1 × 100 mm, kept at 65 °C. LNP samples were diluted 10× with 60 mM Ammonium Acetate (Honeywell, Cat# 14267–25G) in Isopropanol (HPLC grade, Fisher Cat # 67–63–0), then spun down in a microcentrifuge at 14,000x g for 30 min at 4 °C. The supernatant was discarded, and the pellet was washed with Isopropanol. After spinning down again at 14,000x g for 15 min, the supernatant was discarded. The wash steps were repeated one more time, then the samples were put in a vacuum desiccator for approximately 2 h to evaporate off all remaining Isopropanol. The remaining pellet was reconstituted with 100 μL of mobile phase A, then injected on the HPLC system. Injection volume was 5 μL per sample.

#### pH Measurement

pH measurements were taken using a Mettler Toledo SevenCompact pH meter. Calibration was performed day of measurement utilizing a 3-point calibration.

#### SAXS Measurements

Small-angle X-ray scattering (SAXS) measurements were conducted at the Cornell High Energy Synchrotron Source (CHESS) beamline 7A, Argonne National Laboratory (ANL) beamline 121D, and SLAC National Accelerator Laboratory beamline 4–2, employing an Eiger 4 and 9 M detectors. Data were collected an incident X-ray energy of 13 keV and with a sample-to-detector distance of 1.7 m for CHESS, and 3.5 m for ANL and SLAC. Samples were introduced into quartz capillaries with a diameter of 1.55 mm and a wall thickness of 10 μm. LNPs were concentrated to a final RNA concentration of 0.2 mg/mL using Amicon Ultra centrifugal filters (100 kDa MWCO, MilliporeSigma). Samples were kept in their associated temperatures before the measurement, and freeze–thaw samples were thawed on bench before the SAXS experiment. 40 μL of sample was loaded using an automated liquid handling system to ensure reproducibility and to minimize radiation-induced structural alterations. LNP SAXS profiles were subtracted from their associated buffers with the exact component concentrations using RAW software, and the subtracted SAXS intensity profiles were processed with Irena for Igor Pro (WaveMetrics).^73^

#### Cryo-TEM Analysis

Cryo-TEM was performed at the both (1) Sauer Structural Biology Laboratory, The University of Texas at Austin, and (2) Lilly Biotechnology Center (LBC) at Eli Lilly and Company in San Diego, CA. mRNA-LNPs were first concentrated to RNA concentration of 0.4 mg/mL using Amicon Ultra centrifugal filters (100 kDa MWCO, MilliporeSigma) in their associated buffers and kept at their corresponding temperatures. Freeze–thaw samples were thawed on the bench from −80 °C before vitrification. Sample vitrification was carried out using a Vitrobot IV system. Aliquots of 3 μL were applied to 200-mesh copper lacey carbon grids with a waiting time of 5–40s, followed by blotting for 3–6 s before plunge freezing. Vitrified grids were maintained at cryogenic temperatures (<−170 °C) until imaging. Data were acquired on a Glacios Cryo-Transmission Electron Microscope (Thermo Fisher Scientific) operating at 200 kV and equipped with a Falcon 4 Direct Electron Detector (Thermo Fisher Scientific). Images at (1) were collected at a dose rate of 1.48 e^–^/Å^2^/s, with an exposure time of 20 s, corresponding to a total electron dose of 30 e^–^/Å^2^, and a calibrated pixel size of 1.9 Å/pixel. Images at (2) were collected on a Glacios cryoTEM at total dose of 50 e^–^/Å^2^ with pixel size of 0.861 Å/pixel. Raw micrographs were subsequently processed using ImageJ to minimize background noise, enhance image contrast, and correct illumination artifacts.

#### Serum-Starved *In Vitro* Transfection and Cell Viability Assays

The mFLuc mRNA-LNP transfection potencies and cytotoxicities in HepG2 cells were evaluated by Bright-Glo Luciferase Assay System (Promega Cat# E2610) and CytoTox 96 Nonradioactive Cytotoxicity Assay Kit (Promega, Cat# G1780), respectively. HepG2 cells were seeded in a white flat-bottom tissue culture-treated 96-well plate at a density of 5 × 10^4^ cells/well and incubated at 37 °C in a 5% CO_2_ incubator overnight to allow cell adherence. The outer edge wells of the plate were filled with 100 μL of ultrapure distilled water (Invitrogen Cat# 10977–015) to minimize edge effects. On the day of transfection, the mRNA-LNPs were removed from storage and warmed to/thawed at room temperature. The mRNA-LNPs, lipofectamine MessengerMAX (Invitrogen Cat# LMRNA003) encapsulated mFLuc mRNA (positive control), and carrierless mFLuc mRNA (negative control) were diluted to an mRNA concentration of 2.5 ng/μL with 1X Opti-MEM I reduced serum media (Thermo Fisher Scientific Cat# 31985062) supplemented with 1% fetal bovine serum (Cytiva, Cat# SH30071.03). The old transfection media was discarded from the 96-well plate, and the cells were washed with 100 μL of 1X PBS before being replaced with 100 μL of mRNA-LNP, control, or the transfection media for untreated cells (*n* = 3). The plate was incubated at 37 °C in a 5% CO_2_ incubator for a 24 h transfection period. 45 min before the end of the transfection period, 10 μL of 10X Lysis Solution was added to three wells with untreated cells as a positive control (designated “LDH Max”) for the cytotoxicity assay. After the 24 h transfection period is complete, 50 μL aliquots from each well were transferred from the 96-well white transfection plate to a separate clear flat-bottom 96-well cytotoxicity plate. 50 μL of CytoTox 96 Reagent was added to each aliquot in the cytotoxicity plate and incubated in the dark at room temperature for 30 min. 50 μL of Bright-Glo Luciferase Reagent was then added to each well in the transfection plate and the luminescence was immediately measured on a SpectraMax M5e plate reader (Molecular Devices). After the 30 min incubation period is finished for the cytotoxicity plate, 50 μL of Stop Solution was added to each well and the absorbance at 490 nm was measured on the plate reader.

#### High Serum *In Vitro* Transfection Assays

The LNP transfection potencies were evaluated contemporaneously across three human cell lines—HepG2, HEK293T, and Jurkat—using Bright-Glo Luciferase Assay System (Promega Cat# E2610). The cells were seeded in white flat-bottom tissue culture treated 96-well plates at a density of 5 × 10^4^ cells/well and a total volume of 100 μL/well and incubated at 37 °C in a 5% CO_2_ incubator overnight. Plates containing Jurkat cells were also placed on a rocker set to 40 rpm during the incubation period. The outer edge wells of the plate were filled with 100 μL of ultrapure distilled water (Invitrogen Cat# 10977–015) to minimize edge effects. On the day of transfection, the LNPs were removed from storage and warmed to/thawed at room temperature. The LNPs, lipofectamine MessengerMAX (Invitrogen Cat# LMRNA003) encapsulated mFLuc mRNA (positive control), and carrierless mFLuc mRNA (negative control) were diluted to an mRNA concentration of 10.0 ng/μL with high serum (10% FBS) culture media according to each cell line (see culture media information in Materials and Equipment section). The 96-well plates were removed from the incubator and 25 μL of the diluted LNPs, diluted controls, or media (for untreated cells) were added directly to each respective well (*n* = 3). The plates were returned to the same incubation conditions for a 24 h transfection period. After the 24 h transfection period was completed, 125 μL of Bright-Glo Luciferase Reagent (1:1 ratio with the total volume in each well) was then added to each well in the transfection plate and, after a 2 min incubation at ambient temperature, the luminescence was immediately measured on a SpectraMax M5e plate reader (Molecular Devices).

#### Imaging Assay to Assess Nanoparticle Uptake, Endosomal Escape, and Functional RNA Delivery

The nanoparticle uptake, endosomal escape, and functional RNA delivery is assessed using an engineered mCherry-Gal9 HeLa knock in HeLa cell line (iXCells Biotechnologies, order ID: SO2306582) on the Opera Phenix high-content imaging system. mCherry-Gal9 KI HeLa cells were plated in the inner 60 wells of a black/clear flat bottom TC microplate, 96 well (Corning, 356640) at a density of 2 × 10^4^ cells/well and incubated at 37 **°**C in a 5% CO_2_ incubator overnight to allow cell adherence. The outer wells were filled with 200 μL/well of 1X PBS, pH 7.4 (Gibco, 10010023) to account for potential edge effect. The cells were plated in 100 μL/well complete media with DMEM (Gibco, 11–995–073) 10% FBS (Gibco, 10–082–147) and 1 μg/mL Puromycin (Gibco, A1113803). One hour prior to LNP formulation treatment the cells received a media change with 100 μL/well of complete media with Hoechst (Life Technologies, 34580) at a dilution of 1:10,000. The LNP formulations prepared at a stock concentration of 0.2 mg/mL were removed from storage, thawed to room temperature and diluted in Opti-MEM (Gibco, 11058021) to 10, 25, and 50 ng/μL. The complete media was aspirated from the plate and replaced with 90 μL/well of Opti-MEM supplemented with 1% FBS. Ten μL of the prepared LNP formulations at 10x was then added to the plate according to the plate layout for a working concentration of 500, 250, and 100 ng (n = 2). The positive control, chloroquine (Invivogen, tlrl-chq-4), was resuspended in water and diluted in Opti-MEM to its working concentrations of 100 and 50 μM and directly added to the plate according to the plate layout (n = 3). The plate was then loaded on the Opera Phenix with temperature equilibrated at 37 °C and CO_2_ equilibrated at 3%. The plate was imaged using the 40x water objective in the Hoechst, mCherry, Cy5, and GFP channels. Measurements were taken every 30 min for the first 6 h. and every 60 min for the remaining 43 h.

#### Molecular Dynamic Simulations

All-atom molecular dynamics (MD) simulations were conducted on lipid bilayers composed of LP-01:cholesterol:DSPC = 200:200:40 in aqueous 50 mM NaCl and 50 mM buffer solutions using the CHARMM36m force field^74–77^ and the TIP3P water model.^78^ Force field parameters for LP-01 and its protonated form (LP-01^+^) were generated using the Molecular Modeler module in CHARMM-GUI,^79,80^ and bilayer structures were constructed using a locally modified CHARMM-GUI Membrane Builder.^81,82^ Buffer molecules (citrate, Tris, or histidine) were modeled according to their predominant protonation states at pH 4 or 8 and inserted into the aqueous phase using GROMACS.^83^ Each system underwent energy minimization followed by stepwise equilibration under NVT and semi-isotropic NPT conditions using the velocity-rescale thermostat (278.15 K) and the C-rescale barostat (1 bar). After 300 ns of equilibration, production simulations were performed for 200 ns. Structural analyses included the density distributions of lipids and buffer molecules and hydrogen-bonding interactions between LP-01 and buffer species.

## Supplementary Material



## Notes

DENSS description, peak deconvolution methods and raw data, size, encapsulation efficiency, cryo-TEM, SAXS data, peak deconvolution analysis, mRNA-LNP N/P ratio SAXS and peak analysis, cryo-DSC data, CQAs for 1-month stability data, HPLC for RNA-lipid adduct formation, MD simulation results, excess ionizable lipid phase analysis with cryo-TEM and SAXS (PDF)
