# Supplementary material for: Storage Buffer Composition Impacts Internal Structure, Freeze–Thaw Stability, and Transfection Efficiency of mRNA-Lipid Nanoparticles
Source: ACS Nano. 2026 Jun 12;20(25):17972–87. doi: 10.1021/acsnano.5c22170 (PMC13325854; doi:10.1021/acsnano.5c22170)
Supplement: Supplementary file 1 [file nn5c22170_si_001.pdf]

Supplemental Information

Storage Buffer Composition Impacts Internal Structure, Freeze-Thaw Stability, and Transfection Efficiency of mRNA-Lipid Nanoparticles

Meysam Mohammadi-Zerankeshi[1], Dylan Charland[2†], Keira Donnelly[3†], Geoffrey T. Nash[2†], Jiale Shi[4], Dipak Patil[2], Julia Ennis[2], Kenneth G. Rodriguez[2], Sonia Corba[5], Noah Wambolt[5], Haocheng Chueh[2], Khaled AboulFotouh[1], Mohammed R. Kawelah[6], Younghoon Oh[2], Dennis Yang[2], Ken Qian[2], Qiang Cui[4], Keith P. Johnston[6], Daniel Estabrook[2], Alexander E. Marras[1,3]**

[1] Walker Department of Mechanical Engineering, University of Texas at Austin, Austin, Texas 78712, United States

[2] Eli Lilly and Company, Indianapolis, Indiana 46225, United States

[3] Department of Biomedical Engineering, University of Texas at Austin, Austin, Texas 78712, United States

[4] Department of Chemistry, Boston University, Boston, Massachusetts 02215, United States

[5] Eurofins PSS Insourcing Solutions, LLC, Lancaster, Pennsylvania 17601, United States

[6] McKetta Department of Chemical Engineering, University of Texas at Austin, Austin, Texas 78712, United States

*Corresponding author email: estabrook_daniel@lilly.com; amarras@utexas.edu

† Authors contributed equally

Table of Contents

Section 1. Supplemental Methods 3

Section 2. Fresh mRNA-LNPs with different ionizable lipids and storage buffers CQAs and cryo-TEM 6

Section 3. SAXS data of empty- and mRNA-LNPs with different ionizable lipids and storage buffers 7

Section 4. SAXS peak deconvolution results 8

Section 5. Correlation of peak deconvolution results and transfection efficiency 13

Section 6. Cryo-DSC of mRNA-LNP in different studied storage buffers 14

Section 7. One-month storage of mRNA-LNPs in different storage buffers and temperatures 15

Section 8. The effect of sucrose concentration on internal structure 17

Section 9. The effect of Tris concentration and ionic conditions in the storage buffer 17

Section 10. The effect of Tris molarity on mRNA-lipid adduct formation 21

Section 11. Simulation results on LP-01-cholesterol-DSPC in different storage buffers 22

Section 12. The effect of histidine molarity on CQAs and internal structure of LP-01 mRNA-LNP before and after freeze-thaw 25

Section 13. pH-dependent structural ordering of fresh and freeze-thawed mRNA-LNP with peak deconvolution analysis 26

Section 14. The effect of a pH increment to 8 on LP-01 mRNA-LNPs before and after freeze-thaw 32

Section 15. Supplemental References 32

Section 1. Supplemental Methods

Modulated differential scanning calorimetry of the frozen mRNA-LNP formulations

The glass transition temperature of the maximally freeze-concentrated solution (T_g') was measured using a DSC250 differential scanning calorimeter (TA Instruments, New Castle, DE). Approximately, 5 mg of liquid LNP formulations in different buffers (Tris, histidine or citrate) were weighed into aluminum Tzero hermetic pans and sealed with crimped lids. Samples were cooled from room temperature to -90 °C at 3 °C/min, held at -90 °C for 5 min to ensure thermal equilibrium, and then scanned from -90 to 5 °C at a ramp rate of 3 °C/min with a modulation period of 60 s, amplitude of 1.5 °C, and dry nitrogen purge at 50 mL/min. T_g' onset and point of inflection were determined during the heating scan. All mRNA-LNP formulations were in buffers with identical molarity (50 mM), pH (7.4), and NaCl concentration (45 mM) and contained 5% *w/v* sucrose. As a control, the T_g' of the formulation buffers lacking mRNA-LNPs was also determined.

SAXS parameters and peak deconvolution

Peak deconvolution was implemented using OriginPro 2024 software using Lorentz (Lorentzian) line-shape model:

$$I(q) = I_0 \frac{G^2}{(q-q_0)^2 + G^2} + B \qquad \text{Eq. S1}$$

I_0 is the peak amplitude (maximum intensity), q_0 is the peak center position ($Å^{-1}$), Γ is the half-width at half-maximum ($\Gamma = \frac{FWHM}{2}$), and B is a constant background term. The upper and lower bound of the peak were marked for baseline subtraction using the built-in peak and baseline analysis tool in OriginPro. After baseline subtraction, two peaks were assigned at close to the expected peak center of both the mRNA-lipid and the excess-lipid low-q peak. Then, width, area and center of those two peaks were fitted using the built-in Lorentz function.

The correlation length ξ, associated with short-range ordering was calculated from the Lorentzian width $\xi = \frac{2}{FWHM}$ of the main peak corresponding to mRNA-lipid ordered stacks). We introduce the term "ordering parameter", formed from the ratio of the fitted peak amplitude A to the peak width w (taken as the FWHM or fitted width). Peak amplitude (or intensity) reflects the amount and coherence of the scattering species, while peak breadth is a well-established measure of finite-size effects and limited correlation length. Combining these two quantities into a single ratio provides a compact metric that increases when peaks become both stronger and sharper (*i.e.,* when the sample displays greater structural coherence) and decreases when order is lost (peaks weaken or broaden). This approach follows common practice in X-ray/SAXS/WAXS studies that jointly interpret intensity and FWHM to follow ordering, domain size and correlation-length changes. [1]

$$Ordering\ parameter = \frac{Peak\ intensity}{Peak\ width} \times 100 \qquad \text{Eq. S2}$$

MD Simulations

All-atom simulations of a lipid membrane composed of 200 LP-01 lipids, 200 cholesterol and 40 DSPC molecules in water containing NaCl and buffer molecules were generated using the Chemistry at Harvard Macromolecular Mechanics (CHARMM) graphical user interface (CHARMM-GUI) [2–5] and GROMACS [6] molecular dynamics simulation package. The Membrane Builder module in CHARMM-GUI does not directly support membrane construction for novel lipid species that are not included in its library (such as LP-01). Therefore, force field parameters for LP-01 and its protonated state (LP-01$^+$) were generated using the Molecular Modeler tool within CHARMM-GUI. The Membrane Builder in CHARMM-GUI was then downloaded and modified for local use to construct a bilayer containing 200 LP-01 lipids and 200 cholesterol molecules. A physiological salt concentration of 50 mM NaCl was included in all simulations. The force field files for the buffer molecules (citrate [pK_{a1} = 3.13, pK_{a2} = 4.76, pK_{a3} = 6.40], Tris [pKa = 8.1], and histidine [pK_{a1} = 1.8, pK_{a2} = 6.0, pK_{a3} = 9.2]) and their protonation states at pH 4 and pH 8 were generated using the Molecular Modeler tool in CHARMM-GUI. At pH 4, citrate is predominantly present as citrate$^-$, Tris as Tris$^+$, and histidine as histidine$^+$. At pH 8, citrate is predominantly present as citrate^{3-}; the Tris buffer consists of approximately equal amounts of neutral Tris and Tris$^+$; and the histidine buffer is predominantly neutral histidine. 50mM buffer molecules were inserted into the water layers by the function insert-molecules of GROMACS. All molecular interactions were described using the CHARMM36m [7–9] force field, and water was modeled using TIP3P. [10]

All MD simulations were run using a multistage minimization and equilibration process using the GROMACS molecular dynamics simulation package. Non-bonded interactions used the Verlet cutoff scheme throughout. Electrostatics were treated with Particle-Mesh Ewald (PME) [11,12] with a real-space cutoff of 1.2 nm. Lennard–Jones interactions employed a 1.2 nm cutoff. Simulations were run with periodic boundary conditions in all directions. Bond constraints were applied to all bonds involving hydrogens using LINCS. [13] The membrane was initially minimized using a steepest descent algorithm for 1000 steps. The system was then equilibrated in the Canonical (NVT) ensemble for 125 ps, using a simulation time step of 1.0 fs. Temperature control for this simulation used the Berendsen thermostat [14] with a coupling time of 1.0 ps and a simulation reference temperature of 278.15 K. Initial velocities were generated from the Maxwell–Boltzmann distribution at 278.15 K. A second 125 ps NVT stage was then performed at the same temperature and timestep, with restraints partially relaxed and with velocities continued from the previous run. Following NVT equilibration, the system was transitioned to the isothermal–isobaric (NPT) ensemble and equilibrated in a series of semi-isotropic pressure-coupled simulations using the C-rescale barostat [15] (reference pressure 1 bar in the lateral and normal directions, compressibility 4.5×10^{-5} bar^{-1}, coupling time 5.0 ps). This NPT phase began with a 125 ps run under moderate restraints, then proceeded through successive 500 ps runs in which positional and dihedral restraints were systematically reduced and finally removed. Throughout all NPT stages, temperature was maintained at 278.15 K with the same velocity-rescale thermostat, and semi-isotropic pressure coupling allowed independent relaxation of the bilayer plane and membrane normal. Penultimately, a 300 ns equilibration run was performed using a 1 fs timestep. This was followed by a 200 ns production simulation, from which density distributions of LP-01 and buffer

molecules, as well as hydrogen-bonding interactions between LP-01 and buffer molecules, were obtained.

Density from solution scattering (DENSS)

The pair distribution function of the SAXS data was calculated using Raw software and GNOM.[16] DENSS [17] electron density maps were constructed using "Membrane" mode. This was to capture negative contrast/scattering (0 mean = solvent) from lipids. [18] Negative contrast is denoted by pink regions, which correlates to the overall shape of the particle. Red, yellow, green, cyan, and blue signify positive contrast, which represents the location of the mRNA in the particle. The results were demonstrated in external and cross-sectional views.

Section 2. Fresh mRNA-LNPs with different ionizable lipids and storage buffers CQAs and cryo-TEM

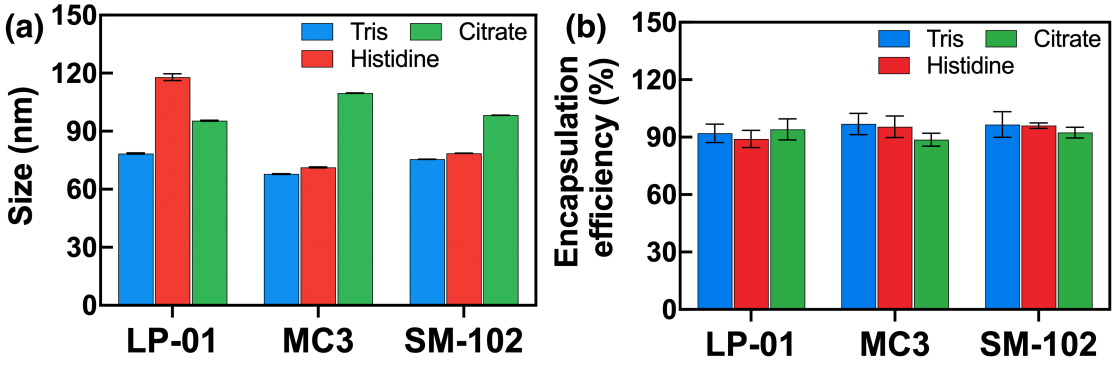

Figure S1. (a) Particle size and (b) encapsulation efficiency of mRNA-LNPs with LP-01, MC3 and SM-102 ionizable lipids in different Tris, histidine and citrate storage buffers.

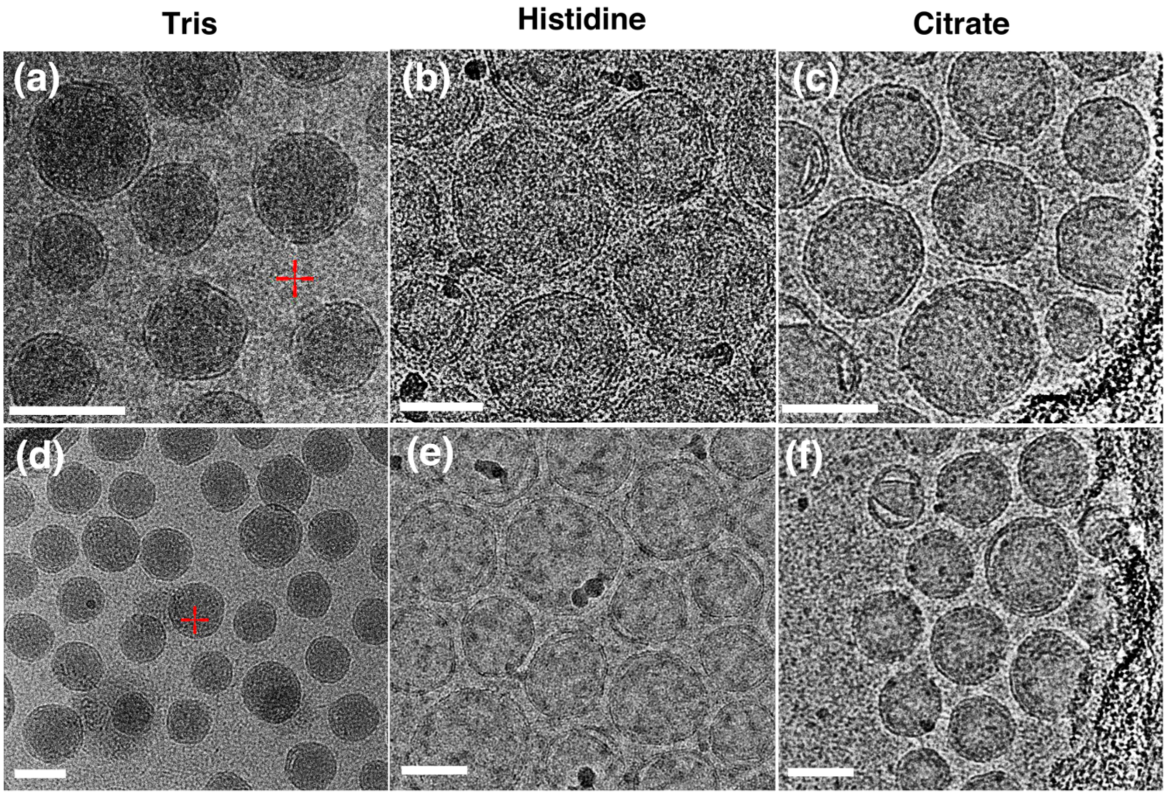

Figure S2. Cryo-TEM images of LP-01 mRNA-LNPs at neutral pH in the studied buffers (a,d) Tris, (b,e) histidine and (c,f) citrate with different magnifications. Scale bars are 60 nm.

Section 3. SAXS data of empty- and mRNA-LNPs with different ionizable lipids and storage buffers

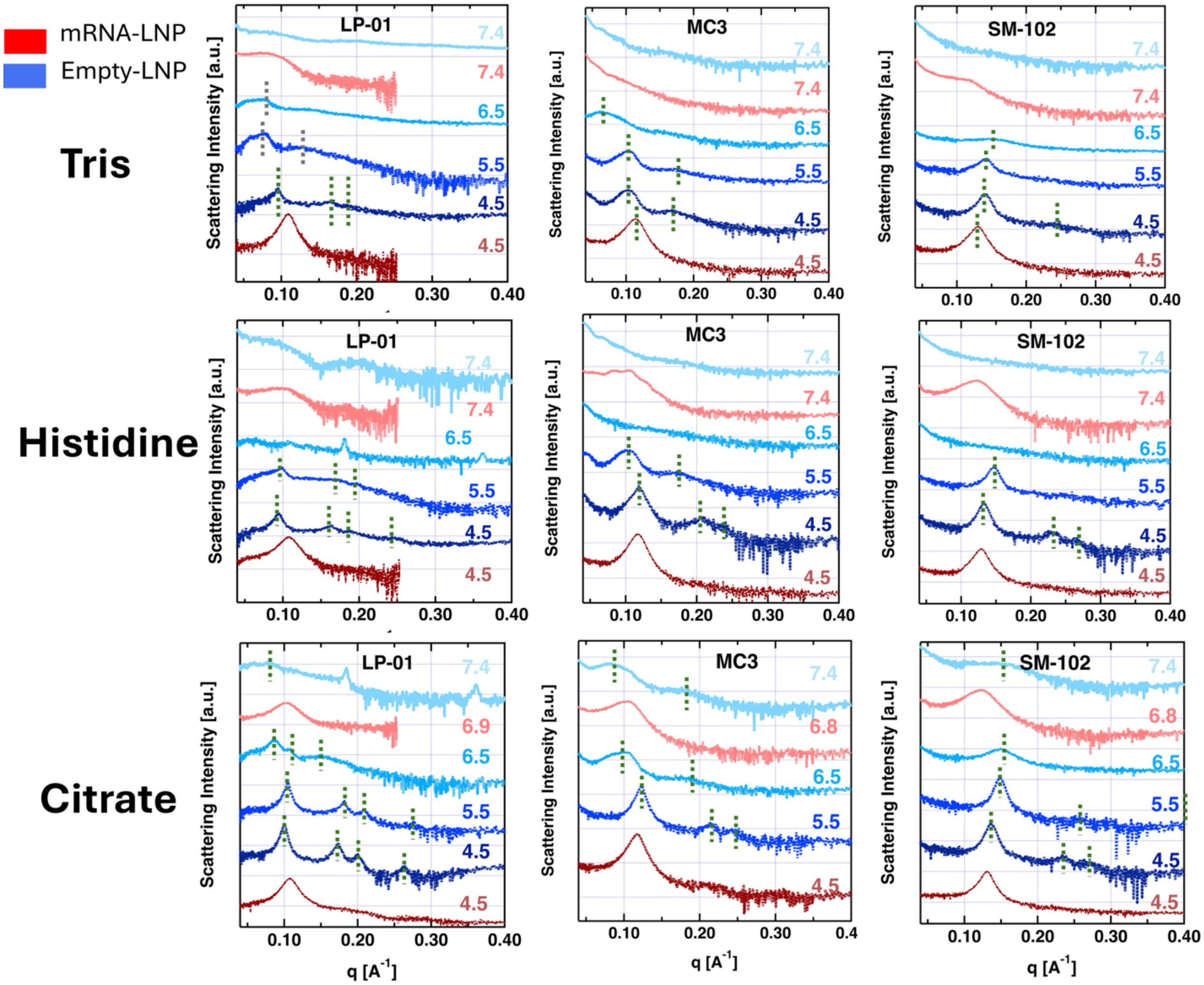

Figure S3. SAXS profiles of empty-LNP (blue) across different pHs from 7.4 to 4.5 and mRNA-LNP (red) at 4.5 and 7.4 pHs with different ionizable lipids and storage buffers.

Section 4. SAXS peak deconvolution results

Using the Lorentz fit, the asymmetrical mRNA-LNPs SAXS data in Figure S3 were deconvoluted into two main mRNA-lipid and excess lipid (or low mRNA) peaks. Peak area, full width at half-maximum (FWHM), peak center, d-spacing (ordering distance), ordering parameter and correlation length for each peak were quantified in Table S1-2. Accordingly, by decreasing the pH, the correlation length increases for the mRNA-lipid and is highest for the most potent LNPs. This trend can be observed for the excess lipid peak. Also, the d-spacing decreases as pH decreases for the mRNA-lipid peak. Excess lipid phase may not exhibit this behavior as the phase would change at different pHs.

Table S1. Fitting parameters of all mRNA-LNPs in Figure 3 for the main mRNA-lipid peak

**Lipid**	**Buffer**	**pH**	**Main peak**						
			**Area**	**FWHM ($Å^{-1}$)**	**Intensity (cm^{-1})**	**Peak center ($Å^{-1}$)**	**d-spacing (nm)**	**Ordering parameter (cm^{-1} Å)**	**Correlation length (nm)**
LP-01	Tris	7.4	1.32E-04	0.0299	0.0028	0.0961	6.5409	9.4220	6.6823
	Tris	4.5	4.83E-04	0.0169	0.0181	0.1089	5.7697	107.1006	11.8343
	Histidine	7.4	1.18E-04	0.0312	0.0027	0.1062	5.9164	8.7500	6.4103
	Histidine	4.5	4.21E-04	0.0214	0.0139	0.1088	5.7755	65.0140	9.3545
	Citrate	6.9	2.53E-04	0.0226	0.0080	0.1077	5.8356	35.1614	8.8456
	Citrate	4.5	3.71E-04	0.0166	0.0155	0.1087	5.7824	93.4139	12.0846
MC3	Tris	7.4	-	-	-	-	-	-	-
	Tris	4.5	4.01E-04	0.0199	0.0140	0.1154	5.4456	70.4523	10.0503
	Histidine	7.4	2.03E-04	0.0260	0.0055	0.1053	5.9664	21.2995	7.6894
	Histidine	4.5	3.54E-04	0.0167	0.0146	0.1188	5.2902	87.6276	12.0120
	Citrate	6.9	1.63E-04	0.0244	0.0050	0.0945	6.6517	20.3279	8.1967
	Citrate	4.5	3.56E-04	0.0135	0.0179	0.1184	5.3085	132.5668	14.8368
SM-102	Tris	7.4	3.69E-05	0.0372	0.0008	0.1000	6.2832	2.0474	5.3763
	Tris	4.5	3.29E-04	0.0204	0.0113	0.1285	4.8893	55.3379	9.7943
	Histidine	7.4	3.15E-04	0.0420	0.0059	0.1162	5.4077	13.9900	4.7585
	Histidine	4.5	3.73E-04	0.0202	0.0130	0.1271	4.9427	64.6154	9.9256
	Citrate	6.9	2.60E-04	0.0337	0.0078	0.1153	5.4499	22.9970	5.9347
	Citrate	4.5	3.92E-04	0.0158	0.0170	0.1300	4.8347	107.3910	12.6342

Table S2. Fitting parameters of all mRNA-LNPs in Figure 3 for the excess lipid peak

Lipid	Buffer	pH	Secondary peak						
			Area	FWHM ($Å^{-1}$)	Intensity (cm^{-1})	Peak center ($Å^{-1}$)	d-spacing (nm)	Ordering parameter (cm^{-1} Å)	Correlation length (nm)
LP-01	Tris	7.4	1.15E-04	0.0261	0.0028	0.0777	8.0885	10.8087	7.6658
	Tris	4.5	1.95E-05	0.0114	0.0010	0.0948	6.6278	8.7719	17.5439
	Histidine	7.4	7.92E-05	0.0313	0.0019	0.0862	7.2908	5.9725	6.3877
	Histidine	4.5	8.67E-05	0.0221	0.0028	0.0970	6.4775	12.7438	9.0703
	Citrate	6.9	2.81E-04	0.0274	0.0075	0.0963	6.5273	27.4417	7.2886
	Citrate	4.5	7.49E-05	0.0145	0.0035	0.1000	6.2832	24.2779	13.7552
MC3	Tris	7.4	-	-	-	-	-	-	-
	Tris	4.5	1.72E-04	0.0236	0.0052	0.1045	6.0143	21.9399	8.4710
	Histidine	7.4	5.38E-05	0.0131	0.0028	0.0838	7.4943	21.2052	15.2555
	Histidine	4.5	1.62E-04	0.0185	0.0061	0.1106	5.6810	32.7754	10.7991
	Citrate	6.9	1.94E-04	0.0303	0.0068	0.1086	5.7883	22.2993	6.6072
	Citrate	4.5	1.36E-04	0.0151	0.0062	0.1106	5.6795	40.9543	13.2538
SM-102	Tris	7.4	1.21E-05	0.0231	0.0010	0.1196	5.2522	4.2079	8.6430
	Tris	4.5	3.56E-05	0.0102	0.0023	0.1363	4.6115	23.0315	19.6850
	Histidine	7.4	8.24E-05	0.0269	0.0022	0.1331	4.7203	8.2467	7.4294
	Histidine	4.5	4.93E-05	0.0102	0.0032	0.1327	4.7367	31.6357	19.5886
	Citrate	6.9	1.56E-04	0.0246	0.00452	0.1290	4.8707	18.4039	8.1433
	Citrate	4.5	1.42E-05	0.0059	0.0016	0.1364	4.6064	26.7797	33.8983

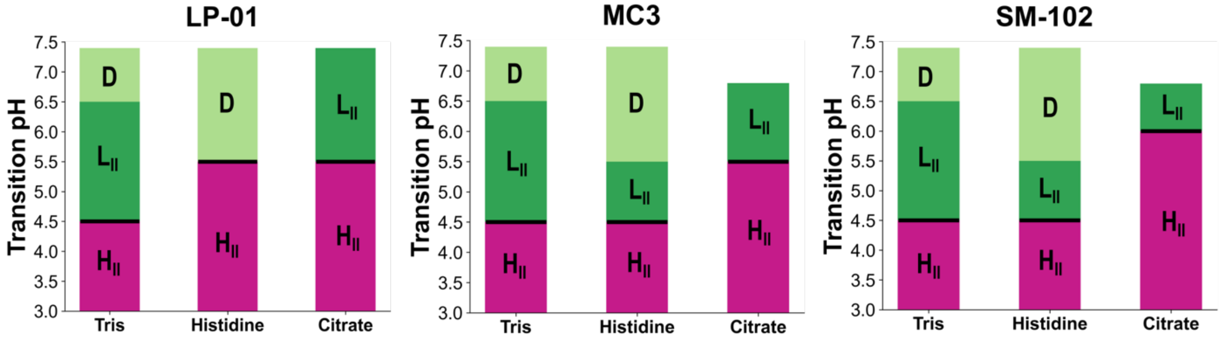

Figure S4. pH-dependent phase transition of empty-LNPs with LP-01, MC3 and SM-102 ionizable lipids in Tris, histidine and citrate storage buffers, obtained from SAXS. Legend: D = disorder; L_{II} = inverse micellar; H_{II} = inverse hexagonal.

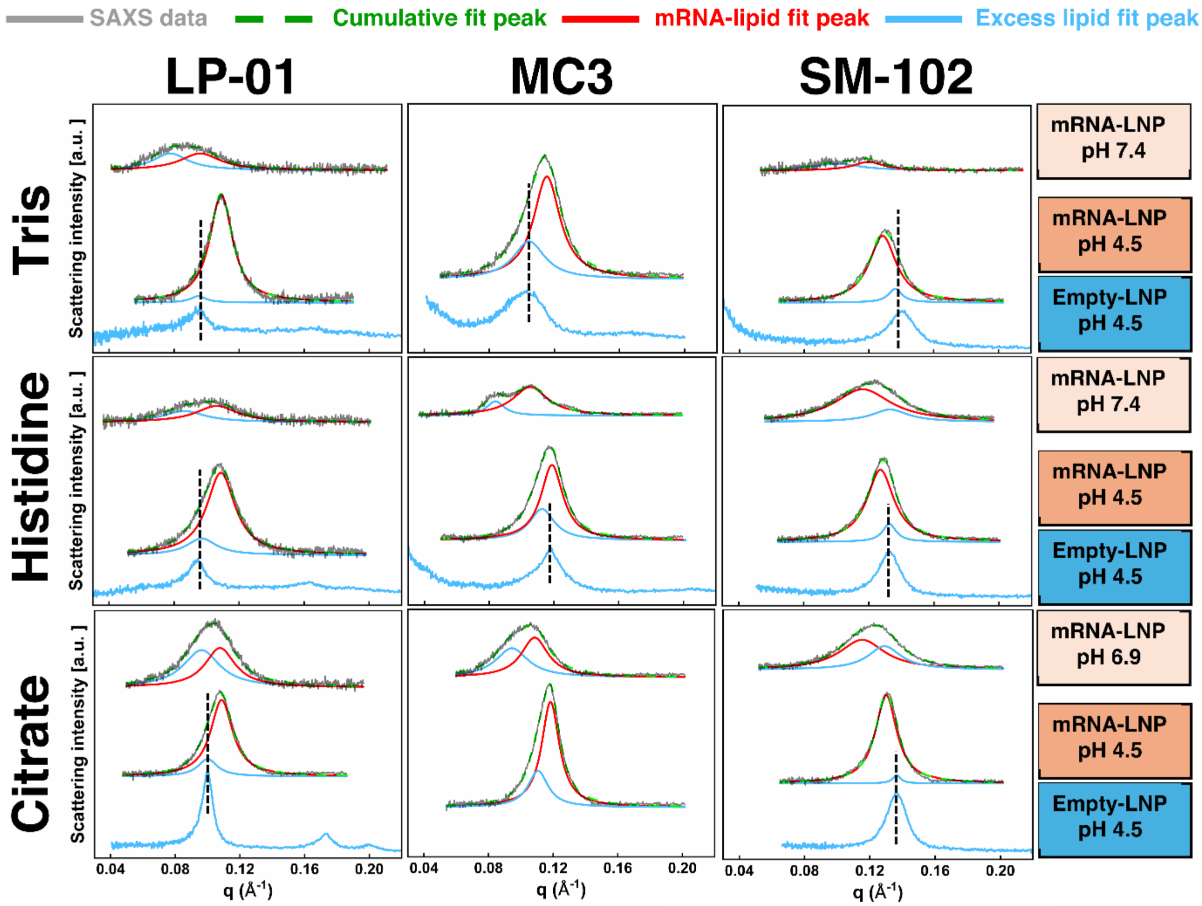

Figure S5. SAXS data fitting using the Lorentz model. The mRNA-LNP at pH 7.4 and pH 4.5 fits with two peaks, corresponding to the mRNA-lipid phase (red fit) and excess ionizable lipid phase (blue fit). The fits show that the excess lipid phase peak in mRNA-LNP aligns with the empty LNP peak at pH 4.5. The bottom blue data is empty LNPs at pH 4.5.

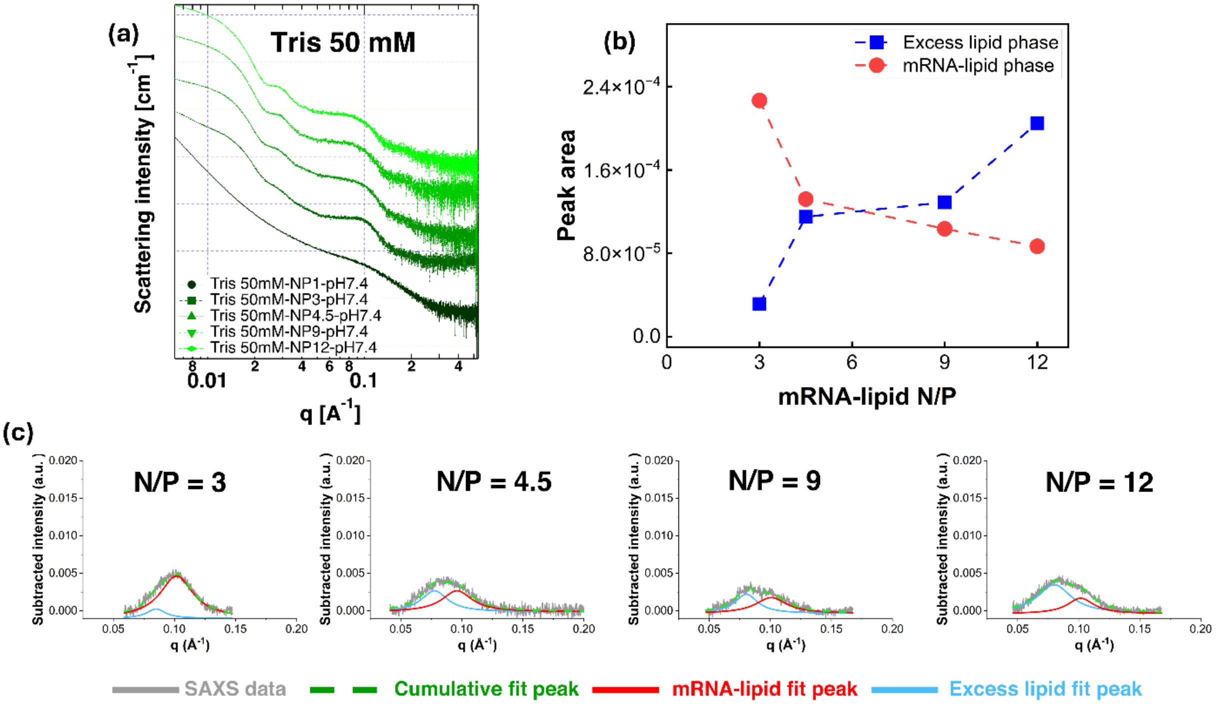

Figure S6. (a-b) SAXS analysis and peak deconvolution of mRNA-LNPs formulated in 50 mM Tris buffer at varying N/P ratios at pH 7.4. (c) Tris 50 mM mRNA-LNP peak deconvolution results in different N/P ratios.

Table S3. Fitting parameters of LP-01 mRNA-LNPs in 50 mM Tris storage buffer at pH 7.4 with different N/P ratios (Figure S6) for the excess lipid peak at fresh conditions.

**Buffer**	**N/P ratio**	**Excess lipid peak**						
		**Area**	**FWHM ($Å^{-1}$)**	**Intensity (cm^{-1})**	**Peak center ($Å^{-1}$)**	**d-spacing (nm)**	**Ordering parameter ($cm^{-1}Å$)**	**Correlation length (nm)**
Tris_50mM	**3**	3.13E-05	0.01878	0.00126	0.085	7.39	6.71	10.65
	**4.5**	1.15E-04	0.0261	0.0028	0.0777	8.09	10.73	7.66
	**9**	1.29E-04	0.0272	0.0029	0.0804	7.81	10.66	7.35
	**12**	2.05E-04	0.0337	0.0038	0.08	7.85	11.28	5.93

Table S4. Fitting parameters of LP-01 mRNA-LNPs in 50 mM Tris storage buffer at pH 7.4 with different N/P ratios (Figure S6) for the mRNA-lipid peak at fresh conditions.

**Buffer**	**N/P ratio**	**mRNA-lipid peak**						
		**Area**	**FWHM ($Å^{-1}$)**	**Intensity (cm^{-1})**	**Peak center ($Å^{-1}$)**	**d-spacing (nm)**	**Ordering parameter ($cm^{-1}Å$)**	**Correlation length (nm)**
Tris_50mM	**3**	2.27E-04	0.03315	0.00565	0.10176	6.17	17.04	6.03
	**4.5**	1.32E-04	0.0299	0.0028	0.0961	6.54	9.36	6.69
	**9**	1.04E-04	0.03108	0.00212	0.10081	6.23	6.82	6.44
	**12**	8.67E-05	0.02647	0.00209	0.10158	6.19	7.90	7.56

Scattering peaks masking effect:

The LP-01 mRNA-LNP in citrate buffer at pH 4.5 demonstrates weak higher-order scattering peaks that roughly align with respective peaks in the empty-LNP with the same formulation, implying that mRNA can mask the higher-order peaks.

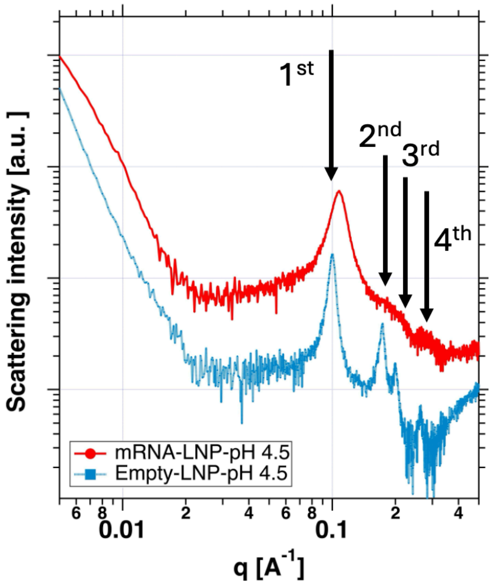

Figure S7. SAXS plot of mRNA-LNP and empty-LNP in citrate buffer at pH 4.5, showing that mRNA disturbs the long-range ordering. In the empty LNP, the intensity of the 2nd-order peak is 24% of the 1st-order peak.

Section 5. Correlation of peak deconvolution results and transfection efficiency

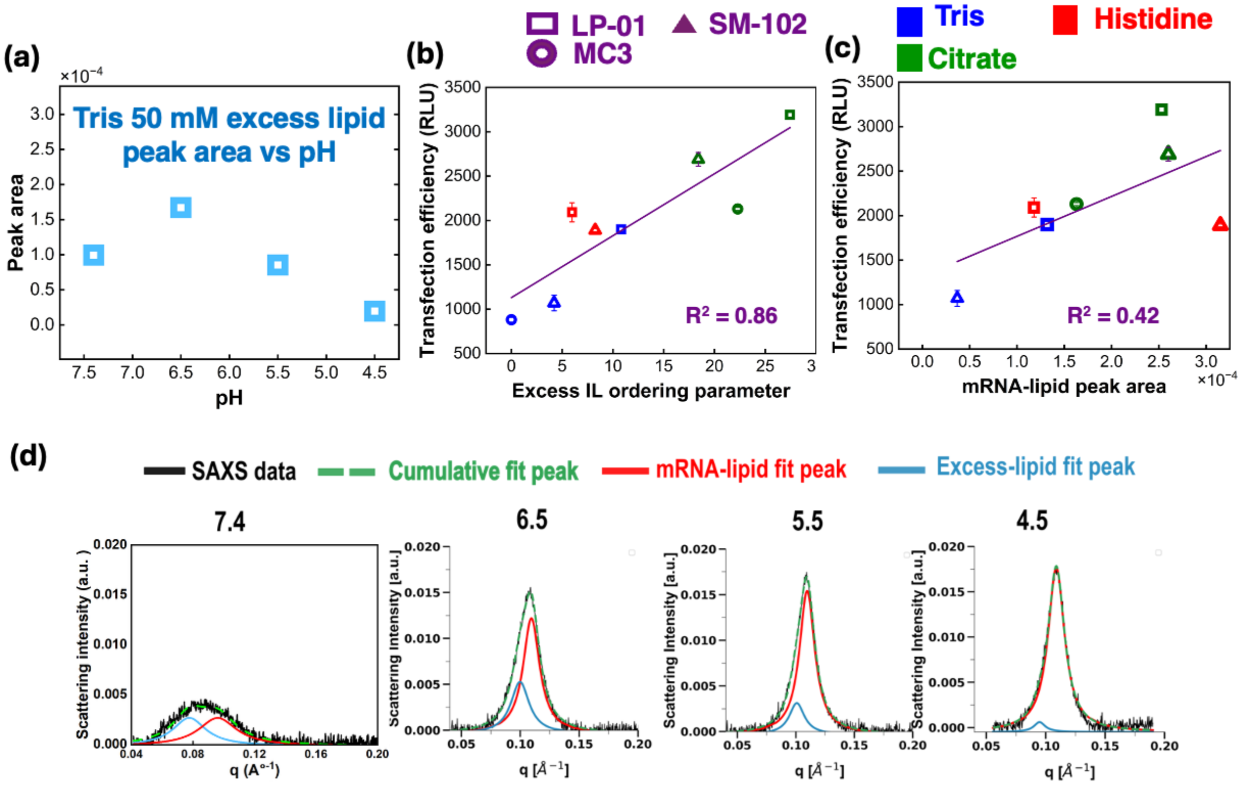

Figure S8. (a) Excess ionizable lipid peak area in different pHs for Tris 50 mM mRNA-LNPs following peak deconvolution. (b) A correlation between transfection efficiency and SAXS ordering parameter (peak intensity/width) for the excess ionizable lipid phase at neutral pH across all buffers and ionizable lipids. Order parameters are further described in Figure S9. (c) A poor correlation between mRNA-lipid peak area and transfection efficiency at pH 7.4, while the excess ionizable lipid peak area showed a strong correlation with transfection efficiency (Figure 3j), highlighting the importance of the excess lipid region. (d) Peak deconvolution plots for 50 mM Tris fresh at different pHs.

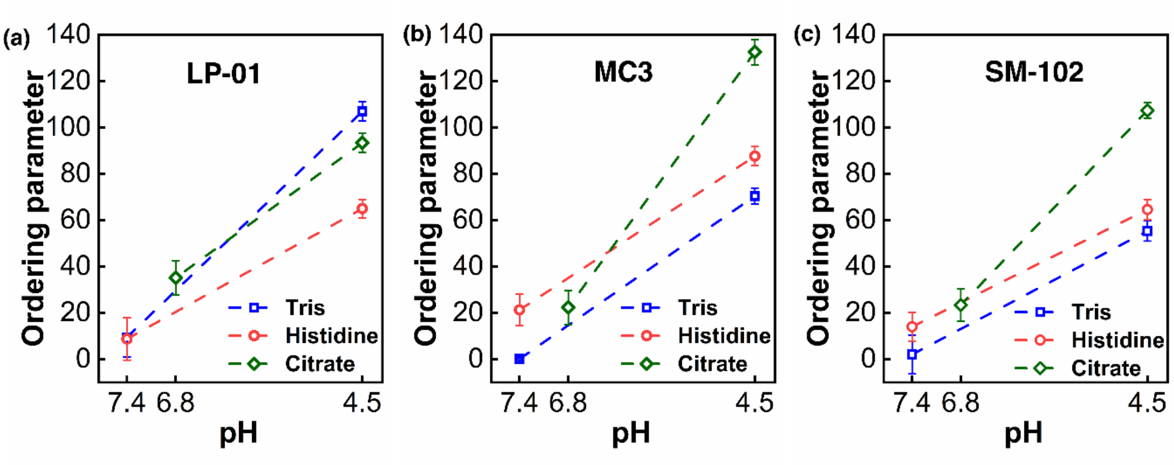

Figure S9. The ordering parameter of the mRNA-lipid phase was calculated by dividing the peak intensity by peak width at half maximum (FWHM), obtained from peak deconvolution of SAXS profiles (Figure S3) in OriginPro software using Lorentz model for mRNA-LNPs in Tris, histidine and citrate storage buffers at high and low pHs with different ionizable lipids: (a) LP-01, (b) MC3 and (c) SM-102.

Section 6. Cryo-DSC of mRNA-LNP in different studied storage buffers

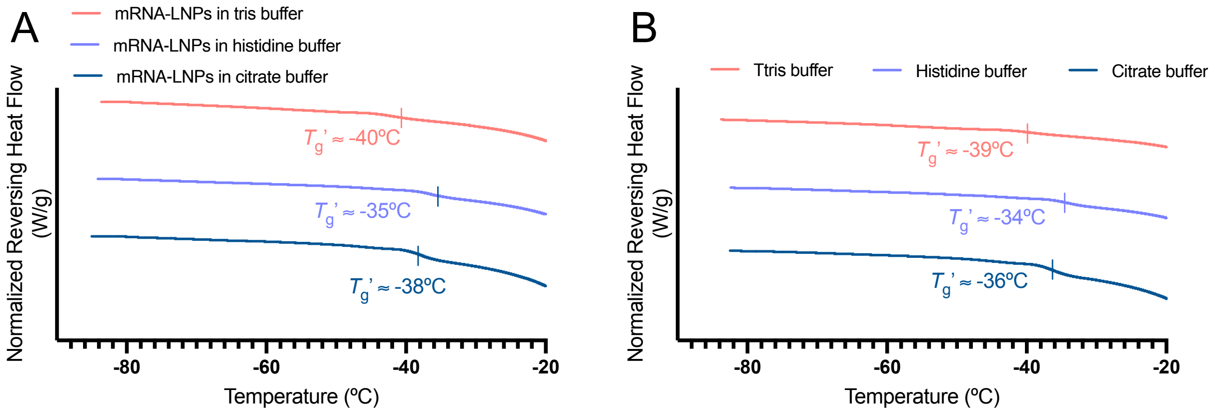

Figure S10. The modulated differential scanning calorimetry thermograms of FLuc LP-01 mRNA-LNPs in different buffers with identical molarity (50 mM), pH (7.4), and NaCl concentration (45 mM) and contained 5% *w/v* sucrose (A) and formulation buffer (without mRNA-LNPs) controls (B).

Section 7. One-month storage of mRNA-LNPs in different storage buffers and temperatures

The SAXS data at 5 ℃ after one month (fresh, before freeze-thaw), before and after freeze-thaw (no long storage) and at -80 ℃ after 1 month show changes in low- and high-q regions. The decrease in the scattering intensity implies the weakened ordering in the internal structure, while the low q region reports on the overall shape of the scattering objects and is very sensitive to structural changes introduced by freeze-thaw cycles. Intact fresh LNPs show a low-q plateau with a slope near zero, which is characteristic of compact spherical particles. For LNPs with structural changes during freeze-thaw, the slope becomes negative and indicates a transition away from spherical symmetry. A slope close to minus one reflects the presence of cylindrical or rod-like structures, which form when spherical particles deform or connect into extended cylindrical assemblies. A slope close to minus two indicates broad two-dimensional features that arise when the original particles lose their curvature and flatten, so the scattering is dominated by large and relatively flat surfaces rather than spherical or cylindrical domains. These slope changes demonstrate that freeze-thaw stress drives a progression from spherical particles to cylindrical objects and finally to flattened structures that no longer behave as discrete nanoparticles in the low q region for citrate-buffered LNPs.

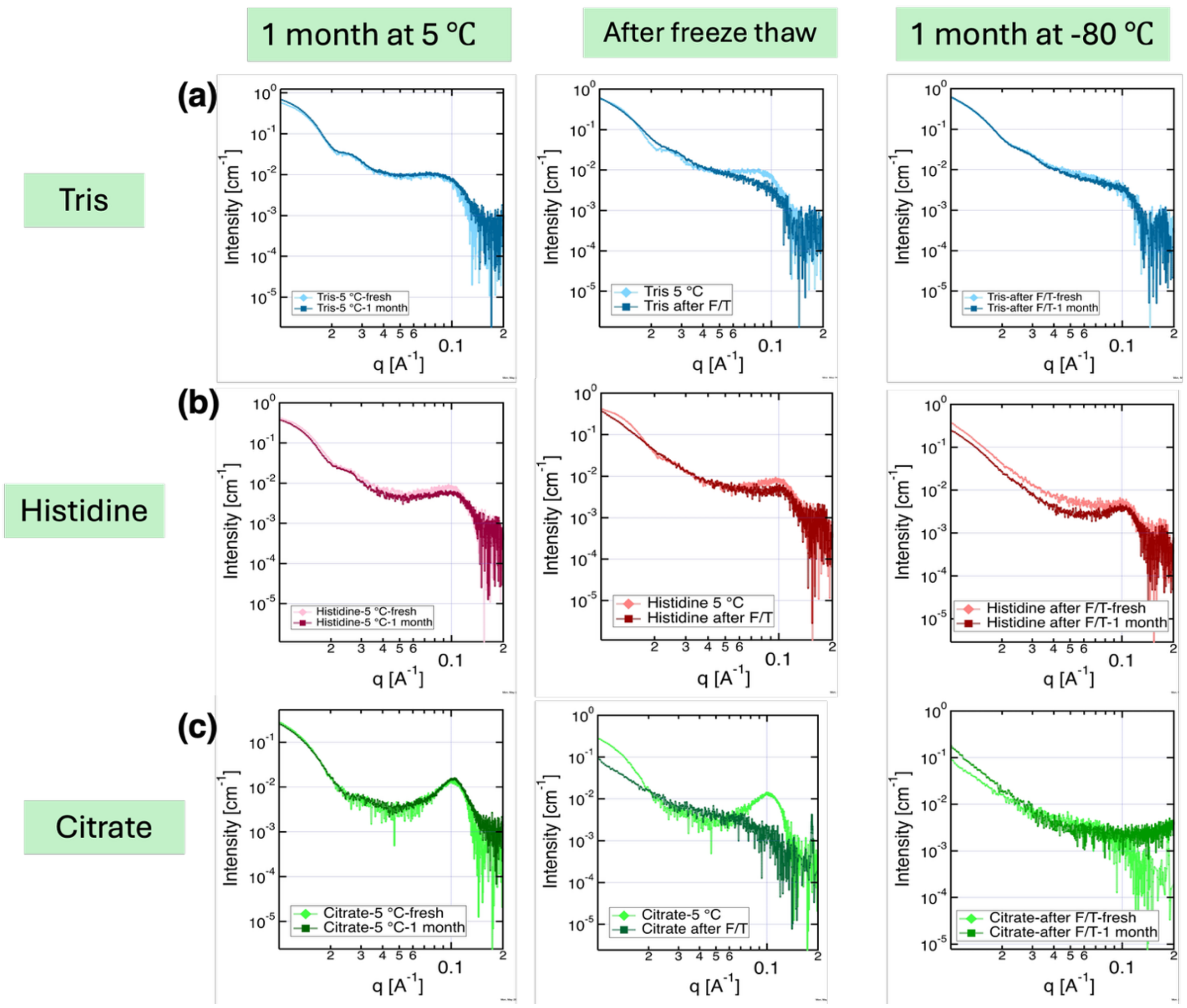

Figure S11. SAXS profiles of LP-01 mRNA-LNPs in different 50 mM buffers, one-month at 5 °C, after freeze-thaw, and one-month at -80 °C (followed by freeze-thaw).

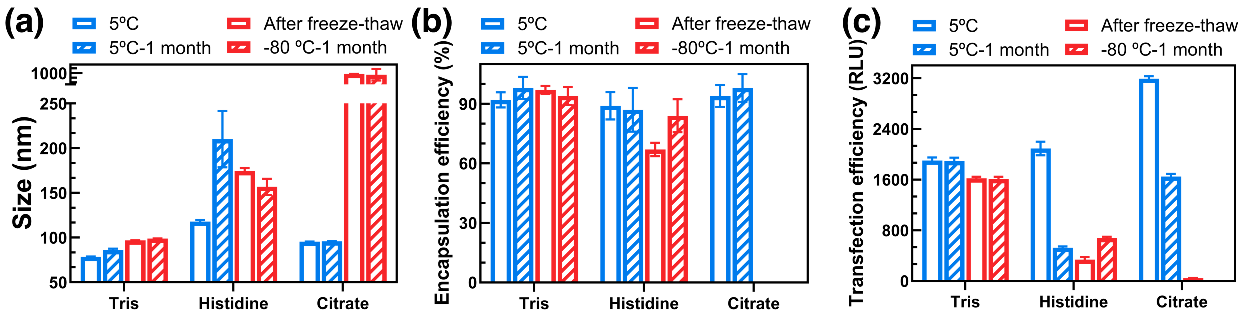

Figure S12. The effect of one-month storage at 5 °C and -80 °C (followed by freeze-thaw) and on size, encapsulation efficiency and transfection efficiency of LP-01 mRNA-LNPs in Tris, histidine and citrate buffers.

Section 8. The effect of sucrose concentration on internal structure

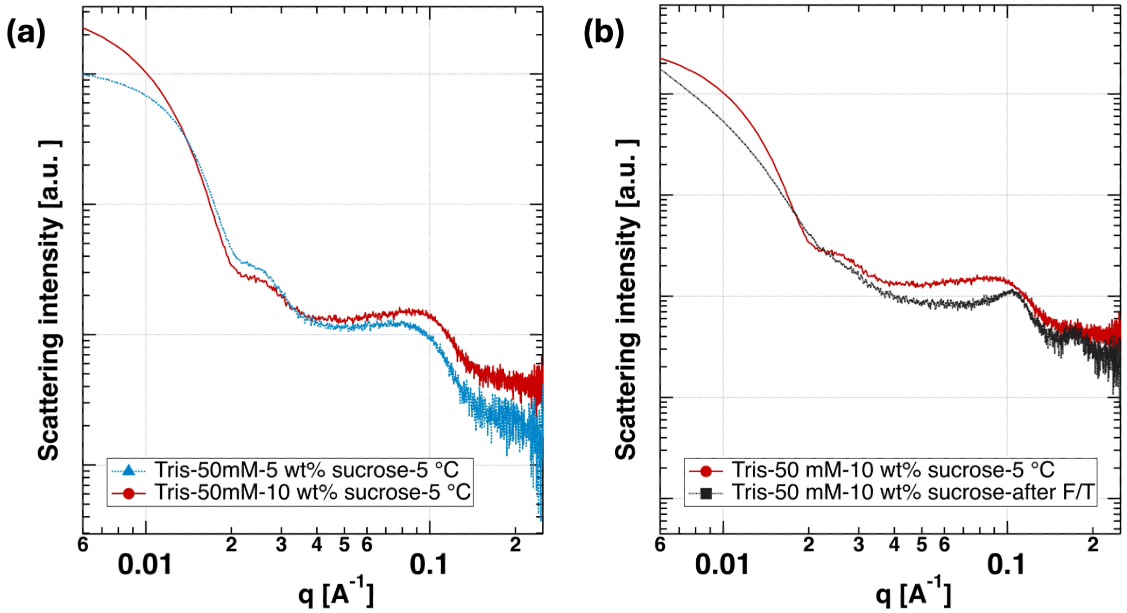

Figure S13. The influence of sucrose content on the scattering profile of 50 mM Tris-buffered mRNA-LNPs at (a) 5 ℃ and (b) freeze-thaw stability.

Section 9. The effect of Tris concentration and ionic conditions in the storage buffer

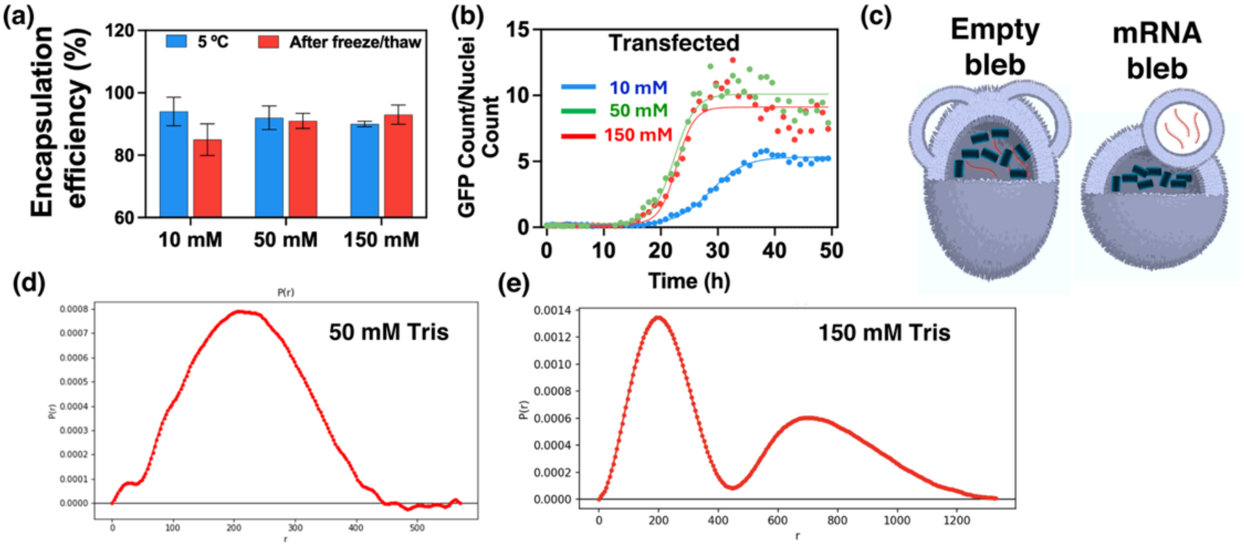

Figure S14. (a) The effect of Tris molarity on the encapsulation efficiency of mRNA-LNPs before and after freeze-thaw. (b) eGFP mRNA transfection results of LP-01 mRNA-LNPs in Tris buffer

with different molarities. (c) A schematic of different types of bleb formation in mRNA-LNPs with different Tris molarity after freeze-thaw. The pair distribution function for fresh mRNA-LNPs in Tris storage buffer with (d) 50 mM Tris and (e) 150 mM, showing asymmetrical P(r) for 150 mM compared to 50 mM Tris formulation due to the blebbed-like morphology.

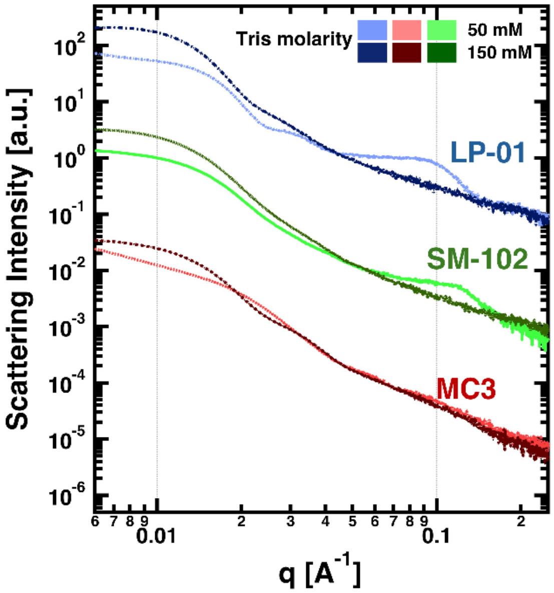

Figure S15. SAXS data of mRNA-LNP with different ionizable lipids in 50- and 150-mM Tris molarities.

(a)

Fluc mRNA-LNPs						After Freeze-Thaw at -80 °C			
							Size	PDI	Encapsulation Efficiency
	Buffer Type	Buffer Molarity (mM)	NaCl (mM)	Sucrose (wt%)	pH	Ionic Strength (mM)	<100 nm	<0.3	>90%
Frozen Buffer Formulations	Tris	10	45	5	7.4	50	125	0.21	85
	Tris	50	45	5	7.4	70	97	0.07	97
	Tris	150	45	5	7.4	120	101	0.06	96
	Tris	10	115	5	7.4	120	161	0.25	86
	Tris	50	95	5	7.4	120	117	0.11	93

(b)

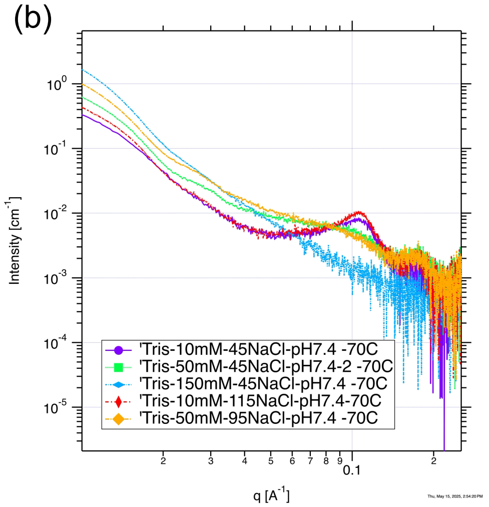

Figure S16. (a) Critical quality attributes for FLuc mRNA-LNPs in buffers with varying concentrations of Tris (10, 50 and 150 mM) and NaCl (45, 95 and 115 mM) after freeze/thaw. (b) SAXS profiles corresponding to FLuc mRNA-LNPs across buffers. Similar ordering is observed between formulations at the same Tris content (*e.g.,* 10 mM Tris + 45 mM NaCl has a similar profile to 10 mM Tris + 115 mM NaCl), but not between formulations at the same ionic strength. These results demonstrate that the disorder in 50- and 150-mM Tris formulations must come from the increased buffer concentration, rather than ionic strength.

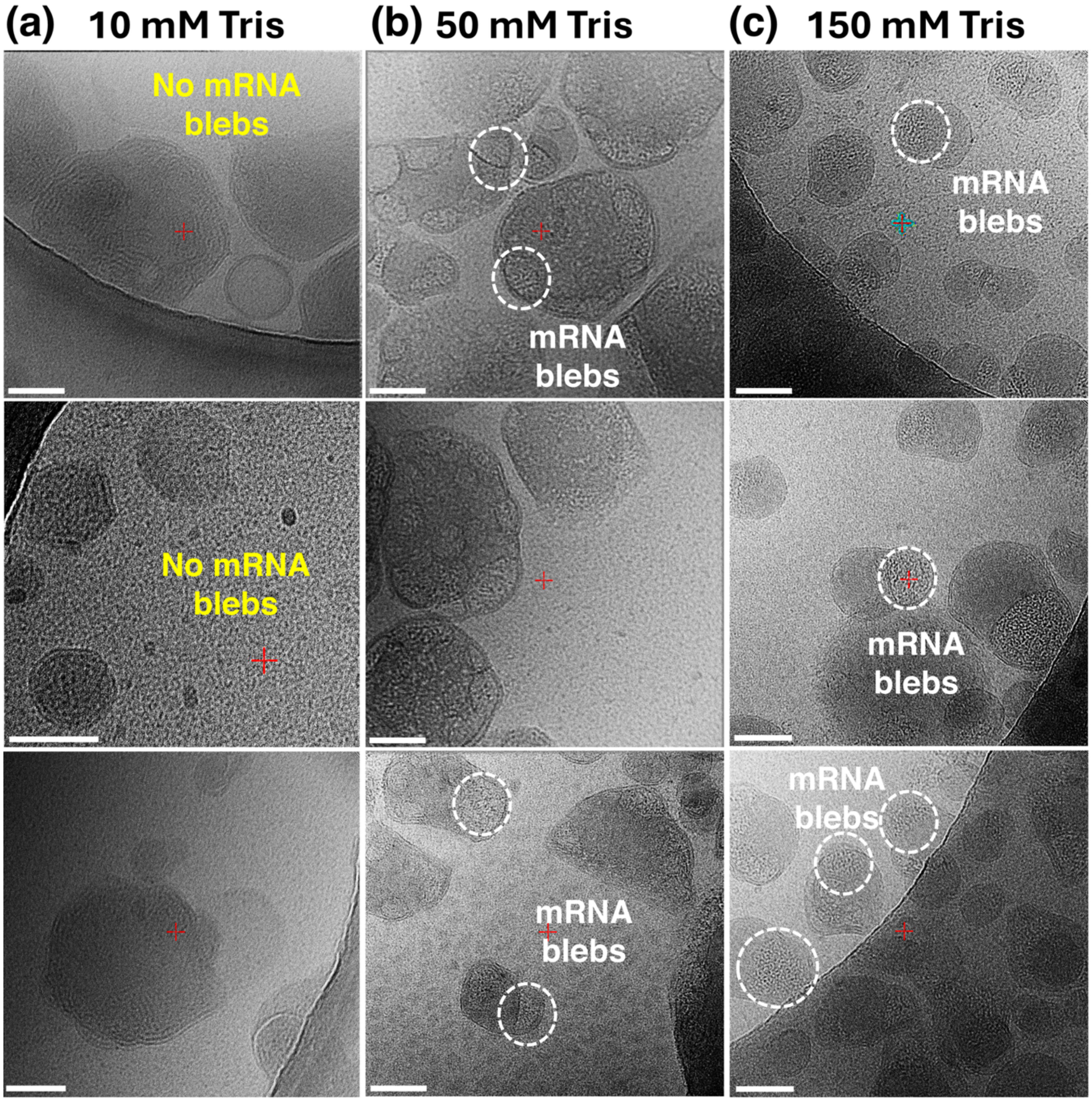

Figure S17. Cryo-TEM images of mRNA-LNP with different Tris molarities: (a) 10, (b) 50 and (c) 150 mM, after freeze-thaw at -80 °C. Scale bars are 60 nm.

Section 10. The effect of Tris molarity on mRNA-lipid adduct formation

(a)

Fluc mRNA-LNPs						1 Month, 5 °C						
						Size	PDI	Encapsulation Efficiency	Transfection (RLU)	RNA Integrity (%)	Late Peak Adduct Formation (%)	Pre-Peak mRNA Fragments (%)
	Buffer Type	Buffer Molarity (mM)	NaCl (mM)	Sucrose (wt%)	pH	<100 nm	<0.3	>90%	>1250 (10^4)	>75%	<10%	<30%
FB Samples	Tris	10	45	5	7.4	116	0.32	91	242	63	19	17
	Tris	50	45	5	7.4	86	0.06	98	2440	83	1	17
	Tris	150	45	5	7.4	88	0.06	96	2990	79	N.D.	21

Buffer Molarity (mM)	1 Month, -80 °C						
	Size	PDI	Encapsulation Efficiency	Transfection (RLU)	RNA Integrity (%)	Late Peak Adduct Formation (%)	Pre-Peak mRNA Fragments (%)
	<100 nm	<0.3	>90%	>1250 (10^4)	>75%	<10%	<30%
10	132	0.20	86	235	72	4	24
50	109	0.07	94	1990	79	N.D.	22
150	101	0.07	95	2410	70	N.D.	30

(b) (c)

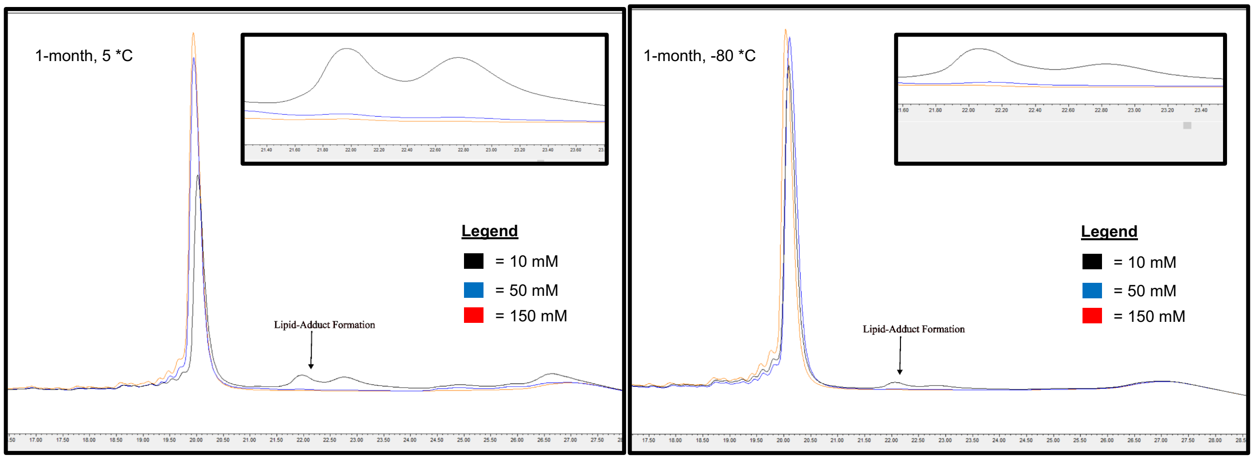

Figure S18. (a) Critical quality attributes for FLuc mRNA-LNPs in buffers with varying concentrations of Tris (10, 50 and 150 mM) after storage for 1 month at 5 °C and -80 °C. (b,c) HPLC analysis of encapsulated FLuc mRNA after 1 month at both (b) 5 °C and (c) -80 °C. Late-eluting peaks are thought to be RNA-lipid adduct species, consistent with the literature.[19]

Section 11. Simulation results on LP-01-cholesterol-DSPC in different storage buffers

Table S5. The charge states of LP-01 and the buffer molecules at pH 4 and pH 8

pH	Neutral LP-01 ratio	Protonated LP-01 ratio	Tris	Histidine	Citrate
**pH 4**	0%	100%	100% $Tris^{+}$	100% $Histidine^{+}$	100% $citrate^{-}$
**pH 8**	100%	0%	50% $Tris^{+}$ and 50% Tris	100% Histidine	100% $citrate^{3-}$

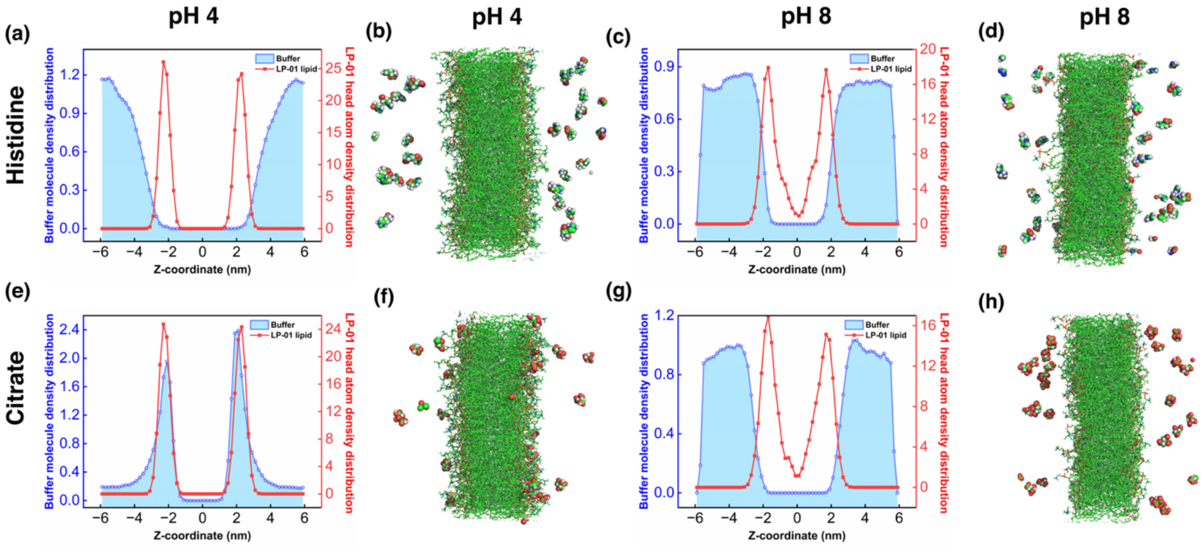

Figure S19. MD simulation results on LP-01-cholesterol-DSPC in (a-d) histidine and (e-h) citrate buffer, highlighting the interaction of buffer molecules with the LP-01 ionizable lipid head atom with storage buffer molecules at pH 4 and pH 8.

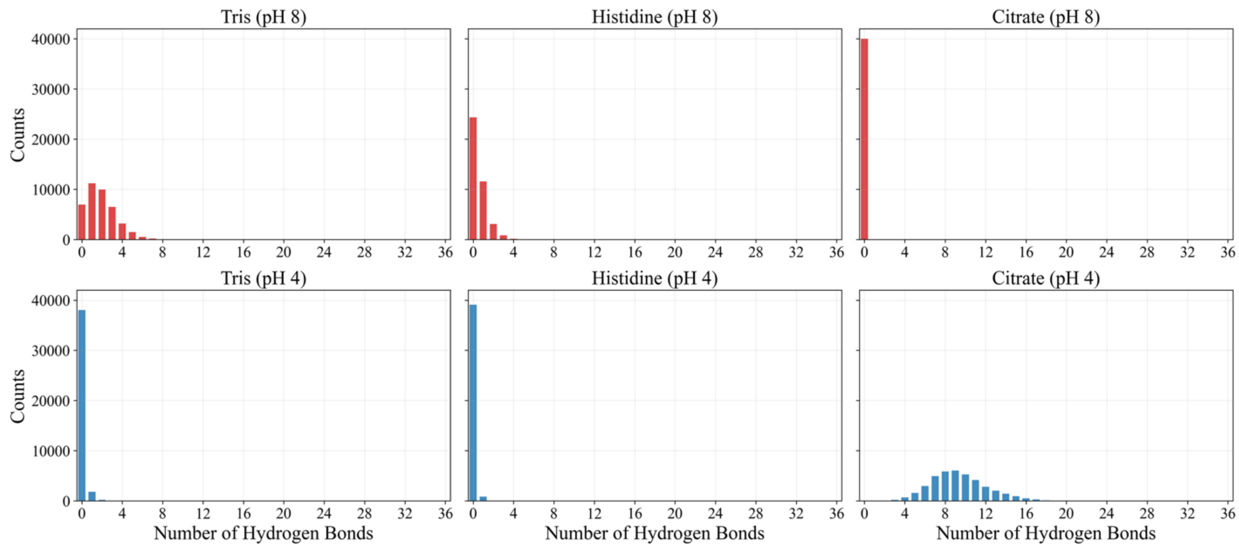

Figure S20. The distribution of hydrogen bonds formed between buffer molecules and ionizable lipids at pH 4 and pH 8, calculated from MD simulations. In the 200 ns production equilibrium molecular dynamics simulation, coordinates were saved every 5 ps, yielding a trajectory of 40,000 frames. In this simulation LP-01, buffer and DSPC are included. For each frame, gmx hbond was used to count the number of hydrogen bonds between the buffer molecules and the LP-01 lipids. At pH 8, the LNP with Tris buffer has the largest number of hydrogen bonds.

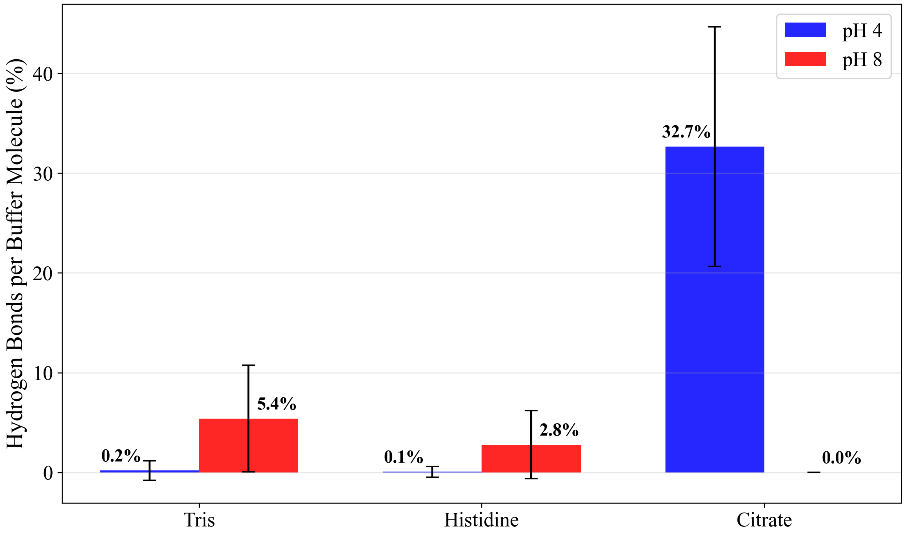

Figure S21. The average number and standard deviation of hydrogen bonds per buffer molecule for 50 mM Tris, histidine, and citrate at pH 4 and pH 8. The standard deviation shows the hydrogen bond distribution deviation from the Gaussian model (Figure S20).

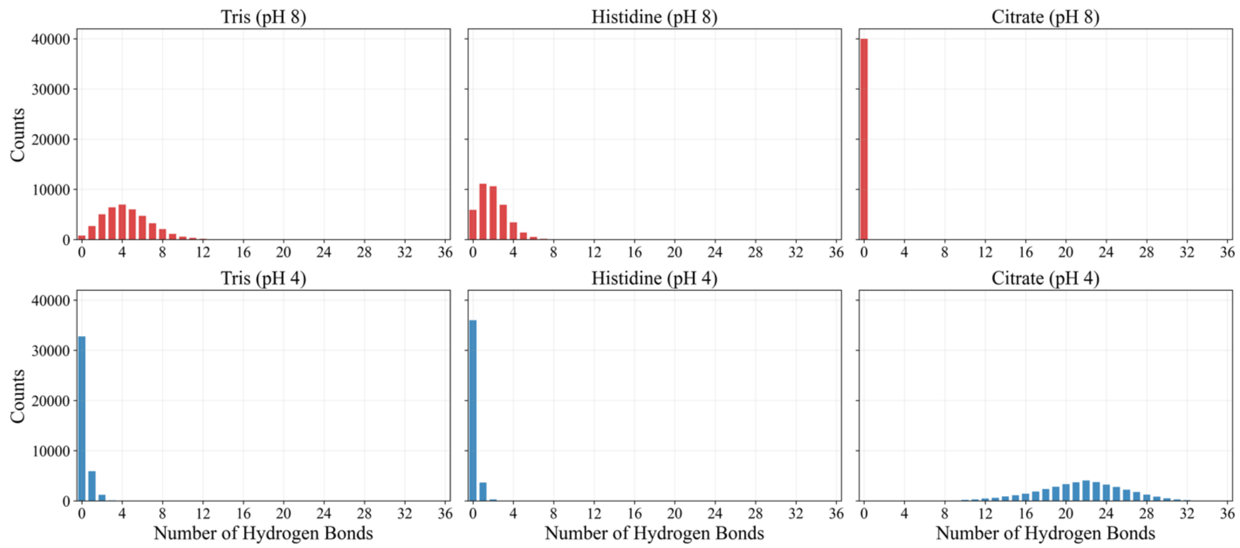

Figure S22. The distribution of hydrogen bonds formed between 150 mM buffer molecules and ionizable lipids at pH 4 and pH 8, calculated from MD simulations. In the 200 ns production equilibrium molecular dynamics simulation, coordinates were saved every 5 ps, yielding a trajectory of 40,000 frames. In this simulation LP-01, buffer and DSPC are included. For each frame, gmx hbond was used to count the number of hydrogen bonds between the buffer molecules and the LP-01 lipids. At pH 8, the LNP with Tris buffer has the largest number of hydrogen bonds.

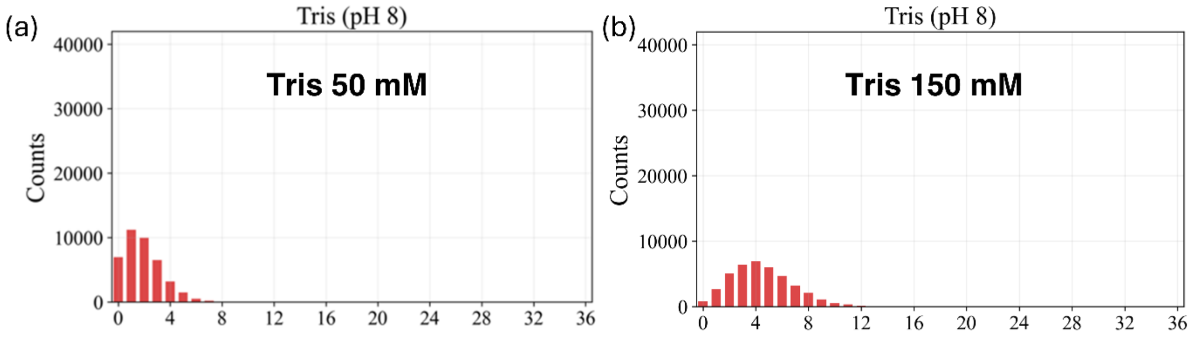

Figure S23. The distribution of hydrogen bonds formed between (a) 50 mM and (b) 150 mM buffer molecules and ionizable lipids at pH 8, calculated from MD simulations. Increasing Tris concentration led to more hydrogen bond formation between buffer molecules and ionizable lipid.

Section 12. The effect of histidine molarity on CQAs and internal structure of LP-01 mRNA-LNP before and after freeze-thaw

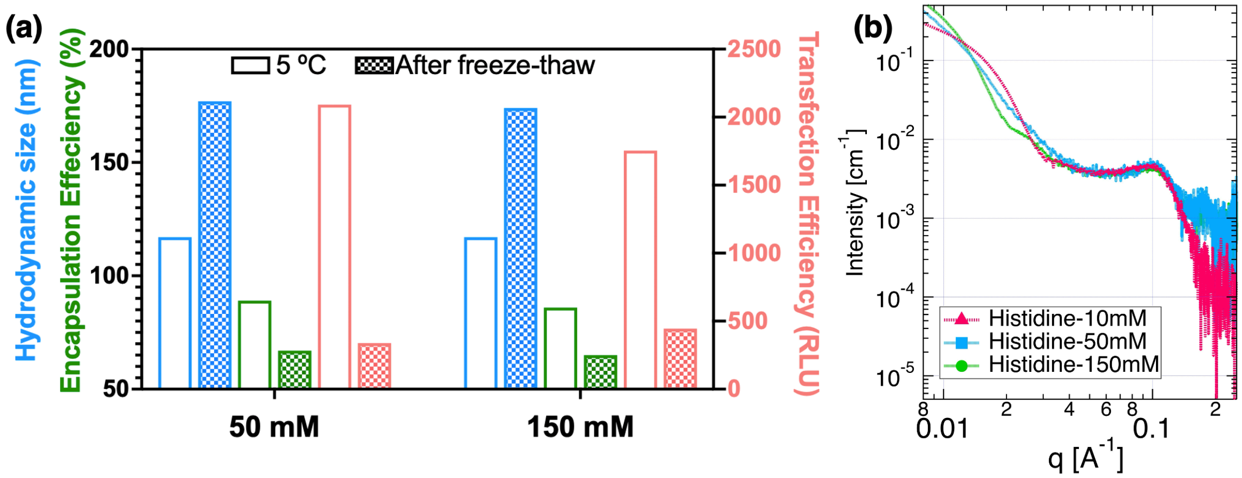

Figure S24. (a) The effect of histidine molarity on size, encapsulation efficiency, and transfection efficiency before and after freeze-thaw. (b) SAXS curves of mRNA-LNPs in histidine buffer with different molarities at 5 °C.

Section 13. pH-dependent structural ordering of fresh and freeze-thawed mRNA-LNP with peak deconvolution analysis

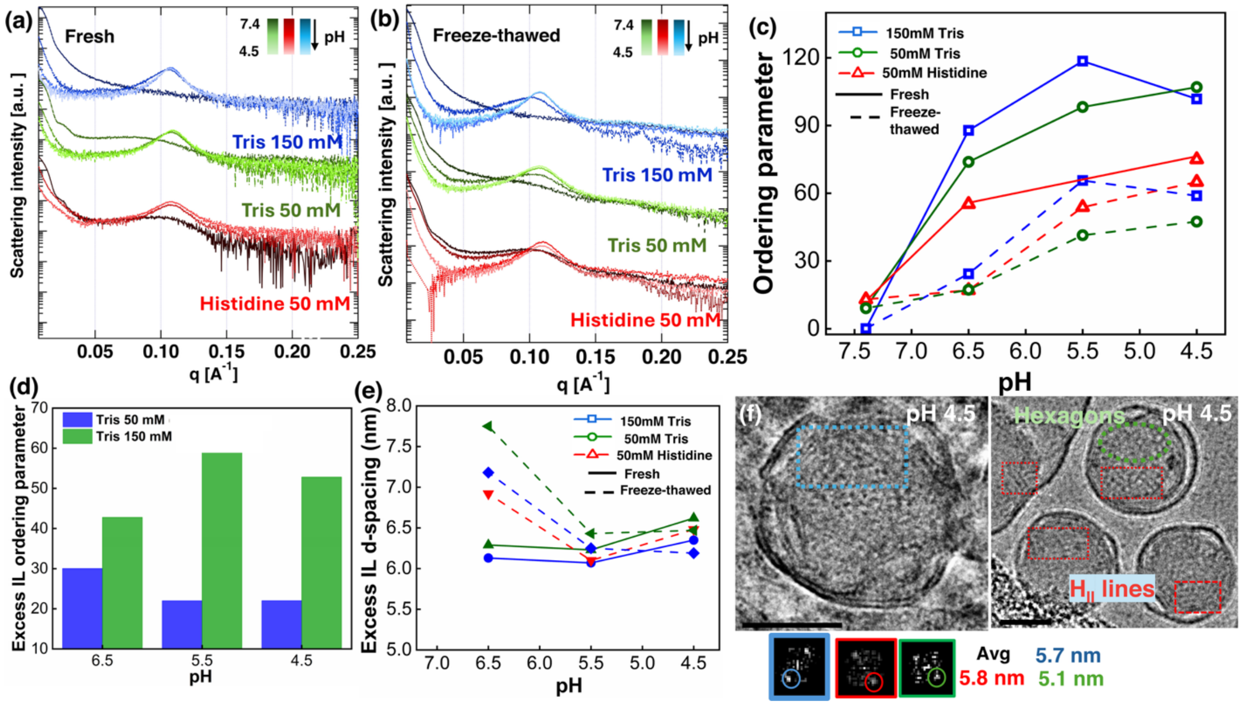

Figure S25. pH-dependent structural changes of mRNA-LNPs to mimic the endosomal pathway (a) before and (b) after freeze-thaw for 50 mM Tris, 150 mM Tris, and 50 mM histidine storage buffers. Here, samples were first freeze-thawed at -80 °C and then pH was reduced. (c) Ordering parameter plot across different pHs obtained from peak deconvolution for the main mRNA-lipid peak emerging by decreasing the pH. (d) excess ionizable lipid (IL) phase ordering parameter for the fresh mRNA-LNPs with 50- and 150-mM Tris molarities. (e) Excess ionizable lipid d-spacing versus pH for fresh and freeze-thawed mRNA-LNPs. (f) Cryo-TEM images of fresh 150 mM Tris-buffered mRNA-LNPs at pH 4.5 show the dominance of the ordered phase. Scale bars = 60 nm.

Table S6. Fitting parameters of LP-01 mRNA-LNPs in Figure S25 for the mRNA-lipid peak before and after freeze-thaw

Buffer	pH	Storage temperature	mRNA-lipid peak						
			Area	FWHM ($Å^{-1}$)	Intensity (cm^{-1})	Peak center ($Å^{-1}$)	d-spacing (nm)	Ordering parameter ($cm^{-1}Å$)	Correlation length (nm)
Tris 50mM	7.4	Fresh	1.32E-04	0.0299	0.0028	0.0961	6.54	9.42	6.68
		FT	7.69E-05	0.0232	0.0021	0.0958	6.56	9.09	8.62
	6.5	Fresh	3.36E-04	0.017	0.0126	0.1094	5.74	73.83	11.74
		FT	1.80E-04	0.0258	0.0044	0.1067	5.89	17.26	7.76
	5.5	Fresh	3.96E-04	0.016	0.0157	0.1097	5.73	98.19	12.47
		FT	2.88E-04	0.0211	0.0087	0.1092	5.76	41.4	9.50
	4.5	Fresh	4.83E-04	0.0169	0.0181	0.1089	5.76	107.10	11.83
		FT	3.14E-04	0.0205	0.0097	0.1095	5.74	47.43	9.75
Tris 150mM	7.4	Fresh	-	-	-	-	-	-	-
		FT	-	-	-	-	-	-	-
	6.5	Fresh	3.00E-04	0.0149	0.0131	0.1076	5.84	87.86	13.42
		FT	2.09E-04	0.0234	0.0057	0.1035	6.07	24.34	8.56
	5.5	Fresh	2.90E-04	0.0126	0.0149	0.1091	5.76	118.52	15.88
		FT	3.06E-04	0.0172	0.0113	0.1086	5.78	65.65	11.60
	4.5	Fresh	2.95E-04	0.0136	0.0138	0.1087	5.78	101.78	14.72
		FT	2.28E-04	0.0157	0.0092	0.1091	5.76	58.91	12.75
Histidine 50mM	7.4	Fresh	1.18E-04	0.0312	0.0027	0.1062	5.92	8.65	6.41
		FT	1.45E-04	0.0267	0.0035	0.1062	5.92	13.00	7.50
	6.5	Fresh	-	-	-	-	-	-	-
		FT	1.48E-04	0.0235	0.004	0.1075	5.84	17.05	8.51
	5.5	Fresh	-	-	-	-	-	-	-
		FT	2.57E-04	0.0174	0.0094	0.112	5.61	53.85	11.47
	4.5	Fresh	4.21E-04	0.0214	0.0139	0.1088	5.78	65.01	9.35
		FT	2.67E-04	0.0206	0.0083	0.1102	5.7	40.05	9.70

Table S7. Fitting parameters of LP-01 mRNA-LNPs in Figure S25 for the excess lipid peak before and after freeze-thaw

**Buffer**	**pH**	**Storage temperature**	**Excess-lipid peak**						
			**Area**	**FWHM (Å^{-1})**	**Intensity (cm^{-1})**	**Peak center (Å^{-1})**	**d-spacing (nm)**	**Ordering parameter (cm^{-1}Å)**	**Correlation length (nm)**
Tris 50mM	**7.4**	Fresh	1.15E-04	0.0261	0.0028	0.0777	8.09	10.81	7.6658
		FT	3.21E-05	0.0167	0.0012	0.0811	7.75	7.36	12.01
	**6.5**	Fresh	1.67E-04	0.0188	0.0057	0.0998	6.29	30.04	10.62
		FT	1.15E-04	0.0294	0.0025	0.0893	7.04	8.48	6.80
	**5.5**	Fresh	8.53E-05	0.0157	0.0035	0.1008	6.23	22.01	12.73
		FT	1.63E-04	0.0260	0.0040	0.0970	6.47	15.31	7.68
	**4.5**	Fresh	1.95E-05	0.0114	0.0010	0.0948	6.62	8.77	17.54
		FT	1.46E-04	0.0224	0.0041	0.0977	6.43	18.54	8.95
Tris 150mM	**7.4**	Fresh	-	-	-	-	-	-	-
		FT	-	-	-	-	-	-	-
	**6.5**	Fresh	1.91E-04	0.0169	0.0072	0.0989	6.35	42.78	11.87
		FT	7.34E-05	0.0212	0.0022	0.0875	7.18	10.36	9.42
	**5.5**	Fresh	1.65E-04	0.0132	0.0079	0.1035	6.07	59.96	15.12
		FT	1.01E-04	0.0198	0.0033	0.1005	6.25	16.53	10.12
	**4.5**	Fresh	1.64E-04	0.0140	0.0074	0.1025	6.13	52.80	14.24
		FT	1.00E-04	0.0162	0.0039	0.1016	6.19	24.34	12.34
Histidine 50mM	**7.4**	Fresh	7.92E-05	0.0313	0.0019	0.0862	7.29	6.07	6.39
		FT	5.08E-05	0.0202	0.0016	0.0894	7.03	7.91	9.89
	**6.5**	Fresh	-	-	-	-	-	-	-
		FT	8.62E-05	0.0270	0.0020	0.0908	6.92	7.54	7.42
	**5.5**	Fresh	-	-	-	-	-	-	-
		FT	1.40E-04	0.0192	0.0047	0.1029	6.10	24.30	10.43
	**4.5**	Fresh	8.67E-05	0.0221	0.0028	0.0970	6.48	12.67	9.07
		FT	4.12E-05	0.0170	0.0015	0.0971	6.47	9.03	11.74

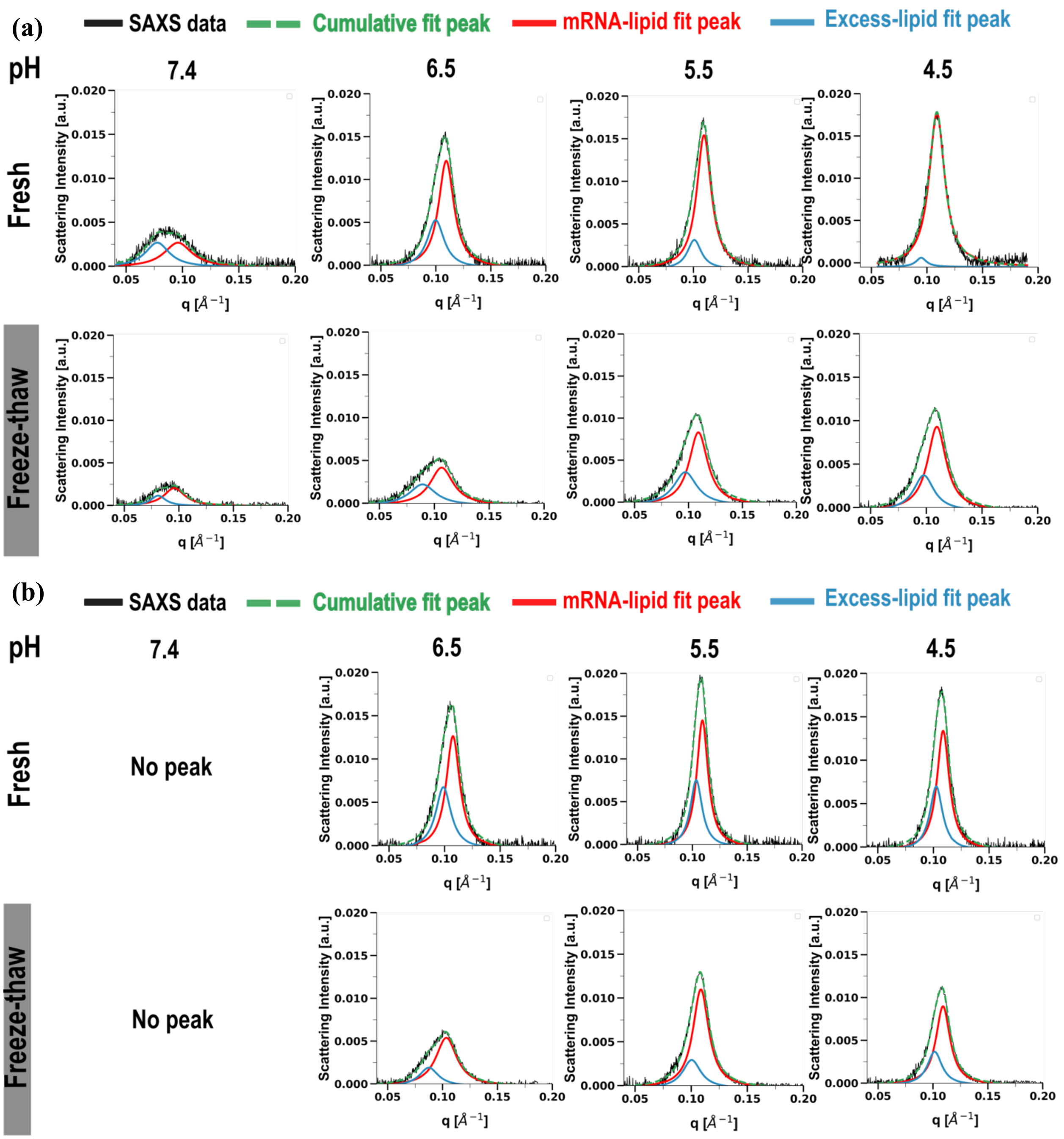

Figure S26. Peak deconvolution of LP-01 mRNA-LNP in (a) 50 mM and (b) 150 mM Tris buffer before (fresh) and after freeze-thaw using two peaks Lorentzian fit.

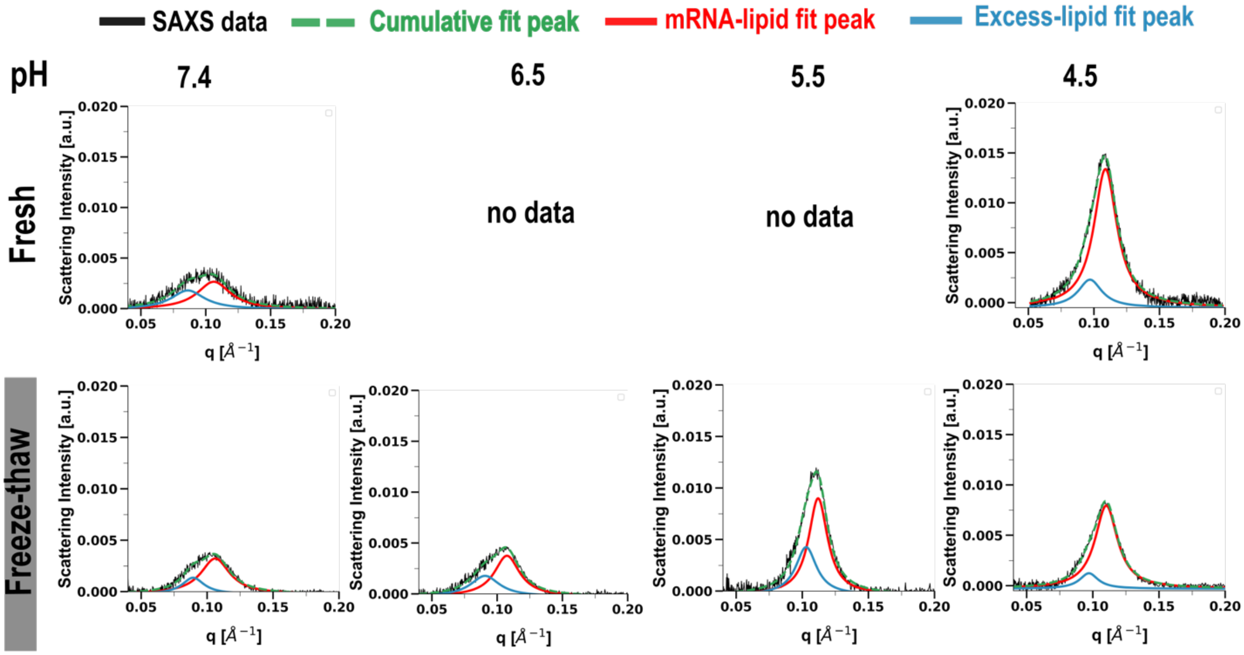

Figure S27. Peak deconvolution of LP-01 mRNA-LNP in 50 mM histidine buffer before (fresh) and after freeze-thaw using two peaks Lorentzian fit.

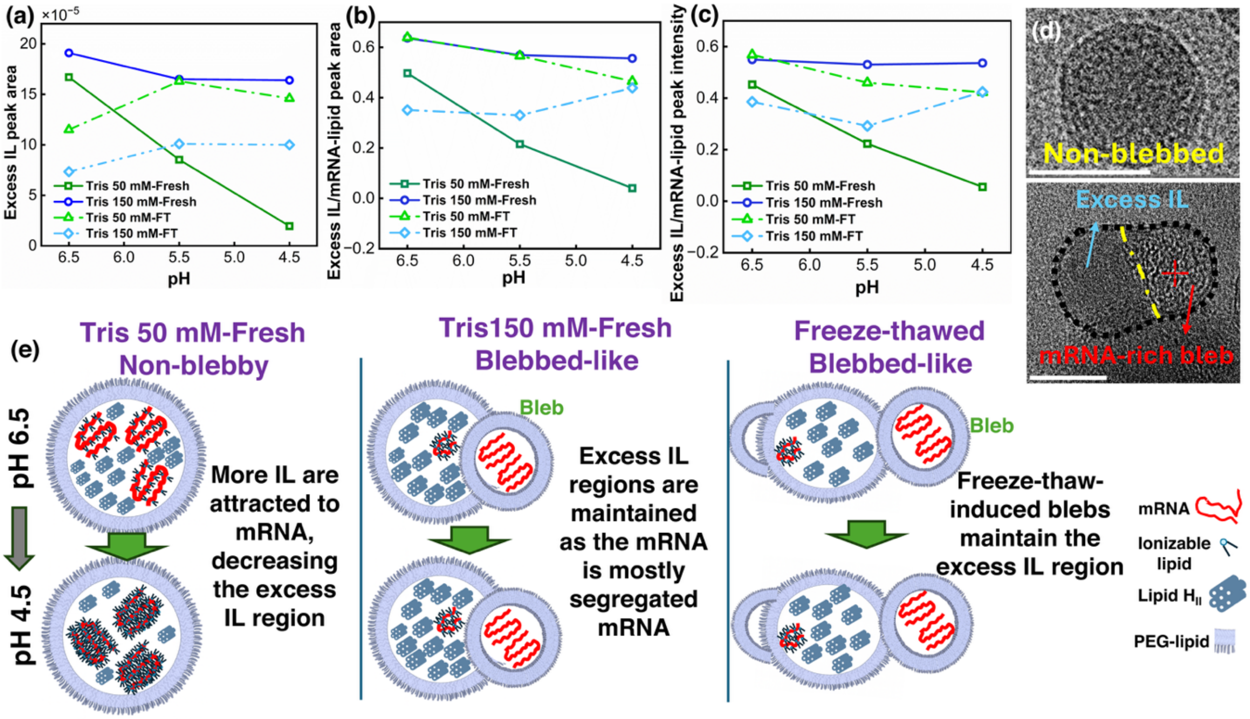

Figure S28. Excess ionizable lipid in blebby and non-blebby mRNA-LNPs. (a) Excess ionizable lipid peak area, (b) the ratio of excess ionizable lipid to mRNA-lipid phase (b) area and (c) intensity of non-blebby Tris 50 mM fresh and blebbed-like fresh Tris 150 mM and freeze-thawed mRNA-LNPs, obtained from peak deconvolution of SAXS results. (d) cryo-TEM of non-blebby fresh Tris 50 mM and Tris 150 mM after freeze-thaw at pH 7.4, showing the mRNA-rich and accessible excess ionizable lipid for H_{II} phase formation. Scale bar is 50 nm. (e) A schematic illustration of the proposed mechanism for non-blebby and blebbed-like mRNA-LNPs during pH decrement.

Table S8. Summary of studied mRNA-LNPs with or without blebs during endosomal pH

Sample	Condition	Bleb	Excess IL phase
**Tris 50 mM**	Fresh	No mRNA-bleb	Decreases with pH decrease
**Tris 50 mM**	Freeze-thawed	mRNA-rich bleb	Maintained with pH change
**Tris 150 mM**	Fresh	mRNA-rich bleb	Maintained with pH change
**Tris 150 mM**	Freeze-thawed	mRNA-rich bleb	Maintained with pH change

Section 14. The effect of a pH increment to 8 on LP-01 mRNA-LNPs before and after freeze-thaw

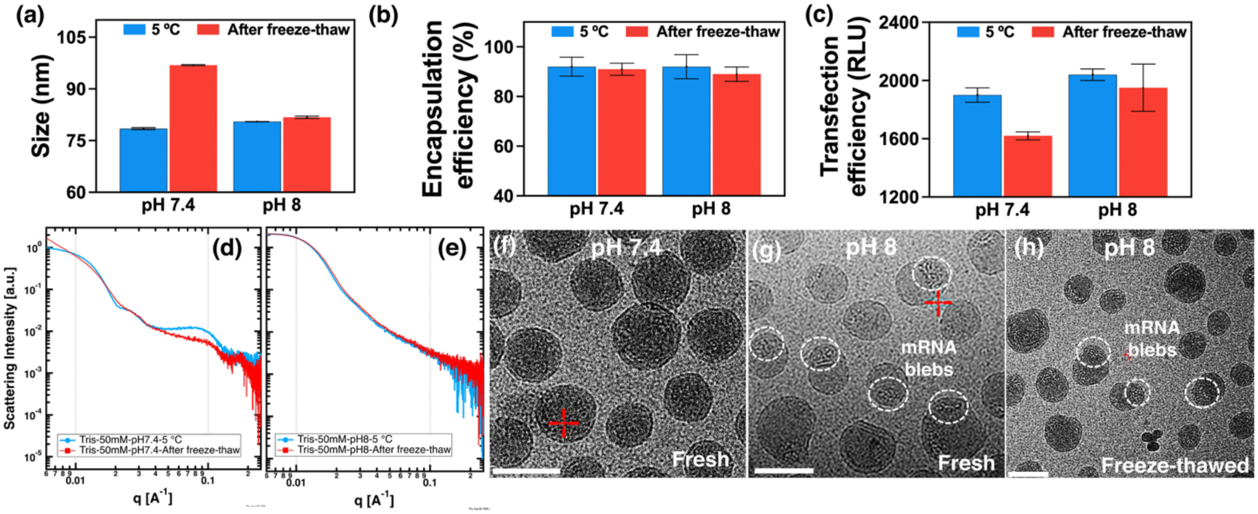

Figure S29. pH increment effect on properties, stability and performance of 50 mM Tris-buffered mRNA-LNP. The influence of increasing the pH of the Tris-buffered mRNA-LNP sample from 7.4 to 8 on (a) size, (b) encapsulation efficiency and (c) transfection efficiency, before (5 °C) and after freeze-thaw at -80 °C. SAXS curves of 50 mM tris-buffered particles in pH (d) 7.4 and (e) 8 before and after freeze-thaw, and cryo-TEM of 50 mM Tris-buffered LNPs at 5 °C in (f) pH 7.4 and (g) pH 8, and (h) Tris pH 8 after freeze-thaw. Scale bars are 60 nm.

Section 15. Supplemental References

(1) Ermrich, M.; Opper, D.; PANalytical (Almelo). *XRD for the Analyst: Getting Acquainted with the Principles*; PANalytical: Almelo, Netherlands, 2013.

(2) Wu, E. L.; Cheng, X.; Jo, S.; Rui, H.; Song, K. C.; Dávila-Contreras, E. M.; Qi, Y.; Lee, J.; Monje-Galvan, V.; Venable, R. M.; Klauda, J. B.; Im, W. CHARMM-GUI *Membrane Builder* toward Realistic Biological Membrane Simulations. *J. Comput. Chem.* **2014**, *35* (27), 1997–2004. https://doi.org/10.1002/jcc.23702.

(3) Park, S.; Choi, Y. K.; Kim, S.; Lee, J.; Im, W. CHARMM-GUI Membrane Builder for Lipid Nanoparticles with Ionizable Cationic Lipids and PEGylated Lipids. *J. Chem. Inf. Model.* **2021**, *61* (10), 5192–5202. https://doi.org/10.1021/acs.jcim.1c00770.

(4) Jo, S.; Kim, T.; Iyer, V. G.; Im, W. CHARMM-GUI: A Web-based Graphical User Interface for CHARMM. *J. Comput. Chem.* **2008**, *29* (11), 1859–1865. https://doi.org/10.1002/jcc.20945.

(5) Kim, S.; Lee, J.; Jo, S.; Brooks, C. L.; Lee, H. S.; Im, W. CHARMM-GUI Ligand Reader and Modeler for CHARMM Force Field Generation of Small Molecules. *J. Comput. Chem.* **2017**, *38* (21), 1879–1886. https://doi.org/10.1002/jcc.24829.

(6) Abraham, M. J.; Murtola, T.; Schulz, R.; Páll, S.; Smith, J. C.; Hess, B.; Lindahl, E. GROMACS: High Performance Molecular Simulations through Multi-Level Parallelism from Laptops to Supercomputers. *SoftwareX* **2015**, *1–2*, 19–25. https://doi.org/10.1016/j.softx.2015.06.001.

(7) Huang, J.; Rauscher, S.; Nawrocki, G.; Ran, T.; Feig, M.; De Groot, B. L.; Grubmüller, H.; MacKerell, A. D. CHARMM36m: An Improved Force Field for Folded and Intrinsically Disordered Proteins. *Nat. Methods* **2017**, *14* (1), 71–73. https://doi.org/10.1038/nmeth.4067.

(8) Choi, Y. K.; Park, S.-J.; Park, S.; Kim, S.; Kern, N. R.; Lee, J.; Im, W. CHARMM-GUI Polymer Builder for Modeling and Simulation of Synthetic Polymers. *J. Chem. Theory Comput.* **2021**, *17* (4), 2431–2443. https://doi.org/10.1021/acs.jctc.1c00169.

(9) Brooks, B. R.; Bruccoleri, R. E.; Olafson, B. D.; States, D. J.; Swaminathan, S.; Karplus, M. CHARMM : A Program for Macromolecular Energy, Minimization, and Dynamics Calculations. *J. Comput. Chem.* **1983**, *4* (2), 187–217. https://doi.org/10.1002/jcc.540040211.

(10) Price, D. J.; Brooks, C. L. A Modified TIP3P Water Potential for Simulation with Ewald Summation. *J. Chem. Phys.* **2004**, *121* (20), 10096–10103. https://doi.org/10.1063/1.1808117.

(11) Darden, T.; York, D.; Pedersen, L. Particle Mesh Ewald: An $N \cdot \log(N)$ Method for Ewald Sums in Large Systems. *J. Chem. Phys.* **1993**, *98* (12), 10089–10092. https://doi.org/10.1063/1.464397.

(12) Essmann, U.; Perera, L.; Berkowitz, M. L.; Darden, T.; Lee, H.; Pedersen, L. G. A Smooth Particle Mesh Ewald Method. *J. Chem. Phys.* **1995**, *103* (19), 8577–8593. https://doi.org/10.1063/1.470117.

(13) Hess, B.; Bekker, H.; Berendsen, H. J. C.; Fraaije, J. G. E. M. LINCS: A Linear Constraint Solver for Molecular Simulations. *J. Comput. Chem.* **1997**, *18* (12), 1463–1472. https://doi.org/10.1002/(SICI)1096-987X(199709)18:12<1463::AID-JCC4>3.0.CO;2-H.

(14) Berendsen, H. J. C.; Postma, J. P. M.; Van Gunsteren, W. F.; DiNola, A.; Haak, J. R. Molecular Dynamics with Coupling to an External Bath. *J. Chem. Phys.* **1984**, *81* (8), 3684–3690. https://doi.org/10.1063/1.448118.

(15) Bernetti, M.; Bussi, G. Pressure Control Using Stochastic Cell Rescaling. *J. Chem. Phys.* **2020**, *153* (11), 114107. https://doi.org/10.1063/5.0020514.

(16) Hopkins, J. B. *BioXTAS RAW 2* : New Developments for a Free Open-Source Program for Small-Angle Scattering Data Reduction and Analysis. *J. Appl. Crystallogr.* **2024**, *57* (1), 194–208. https://doi.org/10.1107/S1600576723011019.

(17) Grant, T. D. Ab Initio Electron Density Determination Directly from Solution Scattering Data. *Nat. Methods* **2018**, *15* (3), 191–193. https://doi.org/10.1038/nmeth.4581.

(18) Grant, T. D. Reconstruction of 3D Density from Solution Scattering. In *Methods in Enzymology*; Elsevier, 2023; Vol. 678, pp 145–192. https://doi.org/10.1016/bs.mie.2022.09.018.

(19) Packer, M.; Gyawali, D.; Yerabolu, R.; Schariter, J.; White, P. A Novel Mechanism for the Loss of mRNA Activity in Lipid Nanoparticle Delivery Systems. *Nat. Commun.* **2021**, *12* (1), 6777. https://doi.org/10.1038/s41467-021-26926-0.
